# Relationship of the Phytochemicals from Coffee and Cocoa By-Products with their Potential to Modulate Biomarkers of Metabolic Syndrome In Vitro

**DOI:** 10.3390/antiox8080279

**Published:** 2019-08-05

**Authors:** Miguel Rebollo-Hernanz, Qiaozhi Zhang, Yolanda Aguilera, Maria A. Martín-Cabrejas, Elvira Gonzalez de Mejia

**Affiliations:** 1 Institute of Food Science Research, CIAL (UAM-CSIC), 28049 Madrid, Spain; 2 Department of Agricultural Chemistry and Food Science, Universidad Autónoma de Madrid, 28049 Madrid, Spain; 3 Department of Food Science and Human Nutrition, University of Illinois at Urbana-Champaign, Urbana, IL 61801, USA; 4 College of Food Science and Biotechnology, Zhejiang Gongshang University, Hangzhou 310000, China

**Keywords:** phytochemicals, inflammation, oxidative stress, adipogenesis, insulin resistance, coffee by-products, cocoa by-products

## Abstract

This study aimed to compare the phytochemicals from coffee and cocoa by-products and their relationship with the potential for reducing markers of inflammation, oxidative stress, adipogenesis, and insulin resistance in vitro. We characterized the phytochemical profile of extracts from coffee husk, coffee silverskin, and cocoa shell and evaluated their in vitro biological activity in RAW264.7 macrophages and 3T3-L1 adipocytes. Pearson correlations and principal component regressions were performed to find the contribution of phytochemicals and underlying mechanisms of action. Coffee husk and silverskin extracts were mainly composed of caffeine and chlorogenic acid. Major components in cocoa shell included theobromine and protocatechuic acid. Both coffee and cocoa by-product extracts effectively reduced inflammatory markers in macrophages and adipocytes (NO, PGE_2_, TNF-α, MCP-1, and IL-6) and the production of reactive oxygen species (21.5–66.4%). Protocatechuic and chlorogenic acids, together with caffeine, were suggested as main contributors against inflammation and oxidative stress. Furthermore, extracts reduced lipid accumulation (4.1–49.1%) in adipocytes by regulating lipolysis and inducing adipocyte browning. Gallic and chlorogenic acids were associated with reduced adipogenesis, and caffeine with adipocyte browning. Extracts from coffee and cocoa by-products also modulated the phosphorylation of insulin receptor signaling pathway and stimulated GLUT-4 translocation (52.4–72.9%), increasing glucose uptake. The insulin-sensitizing potential of the extracts was mainly associated with protocatechuic acid. For the first time, we identified the phytochemicals from coffee and cocoa by-products and offered new insights into their associations with biomarkers of inflammation, oxidative stress, adipogenesis, and insulin resistance in vitro.

## 1. Introduction

According to the World Health Organization, chronic diseases are responsible for 71% of all deaths worldwide [1]. About half of American adults suffer one or more chronic diseases, including cardiovascular disease, obesity, type 2 diabetes, and cancer. These diseases could be prevented through nutrition and adequate food patterns [2]. The disruption of cellular metabolic processes triggers energy and redox imbalance and induces many pathophysiological conditions in the organism, designated as metabolic disorders [3]. The main hallmarks of metabolic disorders include insulin resistance, glucose intolerance, dyslipidemia, and overweight. Likewise, the established chronic oxidative and inflammatory conditions increase the risk of these diseases [4].

Obesity and the resulting metabolic disorders are linked with chronic low-grade inflammation in the adipose tissue [5]. The adipose tissue is an organ with functions of storage, endocrine signaling, and energy homeostasis. Being mostly composed of adipocytes, it also comprises other cell types. Adipose tissue macrophages have been highly associated with the promotion of inflammation and oxidative stress [6]. The promotion of a pro-inflammatory status and oxidative status in the adipose tissue is influenced by the activation of the innate immune system [7]. The adipose tissue produces and releases hormones and cytokines (adipokines) that display different actions on the whole body. Adipokines can elicit the production of reactive oxygen species (ROS), triggering oxidative stress and, consequently, an exacerbated and irregular secretion of other adipokines [8]. The contribution of oxidative stress and mitochondrial dysfunction in cellular and tissue damage in obesity and type 2 diabetes is a hot research topic. A chronic increase in ROS and cytokine levels in adipocytes results in mitochondrial dysfunction, which has a causative role in the attenuation of insulin signaling [9]. Likewise, excessive production of ROS may prompt an inflammatory response through the activation of nuclear factor kappa-light-chain-enhancer of activated B cells (NF-κB) and Jun N-terminal kinase (JNK) pathways [10]. Inflammatory cytokines, primarily secreted by the infiltrated macrophages, induce an exacerbated lipolysis in adipocytes. The deregulated release of free fatty acids activates inflammatory signals in both macrophages and adipocytes, creating an inflammatory interplay between both cell types [11].

Epidemiological and clinical studies have evidenced that dietary phytochemicals improve human health by lowering the risk and preventing chronic diseases. Dietary phenolics are one of the most known natural antioxidant compounds and chemopreventive agents found in food. These compounds are associated with lower risks of obesity, diabetes, and other chronic diseases related with inflammation and oxidative stress [12,13,14]. On the other hand, methylxanthines, such as caffeine and theobromine, have been recognized as effective phytochemicals in weight control, and in the prevention of obesity and type 2 diabetes. Caffeine increases the metabolic rate, energy expenditure, lipid oxidation, and basal-lipolytic and thermogenic activities in the adipose tissue [15,16]. Meanwhile, theobromine has been evidenced as a browning agent that improves lipid catabolic metabolism via the β-adrenergic and AMP-activated protein kinase (AMPK) signaling pathway. Theobromine could exert anti-inflammatory effects; it has demonstrated the ability to reduce cytokines in vitro, in adipocytes and macrophages [17].

Coffee and cocoa by-products, mainly coffee husk, coffee silverskin, and cocoa shell, are produced worldwide in large amounts [18,19]. They are commonly discarded and represent a negative impact on the environment [20,21]. Coffee and cocoa are well-recognized for their health-promoting features [22,23,24]. Their by-products could be considered as potential ingredients for the food and pharmaceutical industries [25,26]. Hence, using these by-products as sources of phytochemicals could be a sustainable strategy in the prevention of chronic diseases. We previously evidenced the modulatory activity of phenolics from coffee and cocoa by-products on obesity-related inflammation in adipocytes and the alleviation of mitochondrial dysfunction and insulin resistance [27,28]. Nonetheless, the bioactivity of extracts from coffee and cocoa by-products has not been compared, nor its association with their phytochemical composition. Hence, this research hypothesized that the different phytochemicals in three plant materials from coffee and cocoa by-products would be related to selected bioactivities. We aimed to compare the phytochemicals from coffee and cocoa by-products and their relationship with the in vitro effects for reducing markers of inflammation, oxidative stress, adipogenesis, and insulin resistance.

## 2. Materials and Methods

### 2.1. Materials

Dulbecco’s modified Eagle medium (DMEM) was obtained from Corning cellgro^®^ (Manassas, VA, USA). Newborn calf serum (NBS), fetal bovine serum (FBS), antibiotic (penicillin–streptomycin, 100×), and 0.25% trypsin–EDTA were purchased from Gibco Life Technologies (Grand Island, NY, USA). All additional reagents were obtained from Sigma-Aldrich (St. Louis, MO, USA) unless otherwise stated.

### 2.2. Preparation and Characterization of Extracts from Coffee and Cocoa By-Products

The coffee husk was provided by “Las Morenitas” (Nicaragua), the coffee silverskin, from Colombia, by Fortaleza S.A. (Spain), both from the Arabica species, and the cocoa shell by Chocolates Santocildes (Castrocontrigo, León, Spain). According to optimized extraction protocols, aqueous extracts were prepared [29]. After milling and sieving, coffee husk (10 g), coffee silverskin (25 g), or cocoa shell (10 g) were added into 500 mL of boiling water (100 °C) and stirred for 90 min for coffee husk and cocoa shell, and 10 min for coffee silverskin. Coffee husk (CHE), coffee silverskin (CSE), and cocoa shell (CAE) aqueous extracts were filtered and frozen at −20 °C for 24 h. Extracts were freeze-dried and stored at −20 °C until further use. The analysis of targeted phenolic compounds was carried following an ultra-performance liquid chromatography electrospray ionization-tandem mass spectrometry in multiple reactions monitoring mode (UPLC–ESI–MS/MS) previously described [30]. Briefly, dissolved extracts were filtered (0.22 μm), and the internal standard 4-hydroxybenzoic-2,3,5,6-d4 acid solution (Sigma-Aldrich, St Louis, MO) (1.25 mg/mL in formic acid/acetonitrile (1:200, v/v)) was added to the samples in a proportion 1:5 (v/v). The column employed was a BEH-C18, 2.1 × 100 mm and 1.7 μm particle size from Waters. The liquid chromatographic system was a Waters Acquity UPLC (Milford, MA) equipped with a binary pump, an autosampler thermostatted at 10 °C, and a heated column compartment (40 °C). The LC effluent was pumped to an Acquity TQD tandem quadrupole mass spectrometer equipped with a Z-spray electrospray ionization (ESI) source. For quantification purposes, data were collected in the multiple reaction monitoring (MRM) mode, tracking the transition of the parent and product ions specific to each compound. The ESI was operated in the negative ionization mode for the analysis of phenolics and in the positive for the analysis of methylxanthines. All compounds were quantified using the calibration curves of their corresponding standards. Injections were carried out in triplicate (*n* = 3).

### 2.3. Cell Cultures

The mouse RAW264.7 macrophages and 3T3-L1 preadipocytes (ATCC, Rockville, MD, USA) cell lines were cultured in DMEM containing 10% (v/v) NBS or FBS, respectively, L-glutamine, sodium pyruvate, and 1% (v/v) antibiotics, and maintained in a humidified atmosphere at 37 °C and 5% CO_2_.

#### 2.3.1. Adipocyte Differentiation

The 3T3-L1 cells were cultured at a density of 6 × 10^3^ cells cm^−2^ and differentiated into adipocytes according to the protocol described by Zebisch et al. [31]. After 48 h, cell differentiation was induced by changing the medium to 10% FBS enriched DMEM containing 0.5 mM 3-isobutyl-1-methylxanthine, 0.25 μM dexamethasone, 1 μg/mL insulin, and 2 μM rosiglitazone. After 48 h, the medium was changed to 10% FBS enriched DMEM containing 1 μg/mL insulin for 48 h. On day 7, the medium was changed to 10% FBS enriched DMEM and the cells were kept in this culture media until they achieve the mature adipocyte phenotype. Cells were fully differentiated at day 10–12 and then employed for the following experiments.

#### 2.3.2. Cell Viability Assay

Cell viability of macrophages, pre-adipocytes, and mature adipocytes treated with extracts (31–500 μg/mL) was performed with the CellTiter^®^ 96 Aqueous One Solution Proliferation assay (Promega Corporation, Madison, WI, USA) following manufacturer’s instructions.

### 2.4. Anti-Inflammatory Potential

The anti-inflammatory effects of extracts from coffee and cocoa by-products were evaluated in both RAW264.7 macrophages and 3T3-L1 mature adipocytes. Macrophages were seeded at a density of 7 × 10^4^ cells cm^−2^ and maintained 24 h in DMEM. Then, macrophages were co-treated for 24 h with lipopolysaccharide (LPS) (1 μg/mL) and the aqueous extracts (31–500 μg/mL) simultaneously. The conditioned media (CM) obtained after the stimulations of macrophages with LPS (1 μg/mL) was used to develop inflammatory conditions in adipocytes. Mature adipocytes (after 10–12 days of differentiation) were incubated for 24 h with CM and the extracts (31–500 μg/mL). Cells and their contact media were collected and stored at −80 °C until analyzed.

#### 2.4.1. Determination of Inflammatory Factors in Macrophages

NO concentration in the cell media was determined using the Griess reagent according to the manufacturer’s protocol and using NaNO_2_ standard curve. Prostaglandin E2 (PGE_2_), tumor necrosis alpha (TNF-α), and monocyte chemoattractant protein 1 (MCP-1) were quantified in the cell culture media using commercially available kits following the manufacturer’s instructions (PGE_2_ ELISA from Cayman Chemical (Ann Arbor, MI, USA) and TNF-α and MCP-1 ELISA kits from R&D systems (Minneapolis, MN, USA)).

#### 2.4.2. Assessment of Adipokines and Inflammation-Triggered Lipolysis in Adipocytes

The amount of TNF-α, MCP-1, interleukin 6 (IL-6), and adiponectin released to the medium was determined using a Milliplex^®^ MAP Mouse Adipocyte Luminex assay (Millipore, Billerica, MA, USA). The culture media was used for glycerol quantification using a glycerol cell-based assay kit, and the cell lysates were assayed for triglycerides (TAG) and lipase activity using colorimetric assay kits. All the kits were purchased from Cayman Chemical.

### 2.5. Antioxidant Potential

The antioxidant activity was also evaluated in RAW264.7 macrophages and 3T3-L1 mature adipocytes. Treatments were conducted as described in Section 2.4. In addition, RAW264.7 macrophages (7 × 10^4^ cells cm^−2^), maintained 24 h in DMEM, were stimulated for 4 h with 100 μM H_2_O_2_ and the extracts were added simultaneously.

#### 2.5.1. Detection of Intracellular ROS, Mitochondrial Superoxide, and Mitochondrial Membrane Potential

After treatments, ROS were measured by incubating the cells for 1 h in DMEM with 2′,7′-dichlorofluorescein diacetate (25 μM). After washing with phosphate buffer solution (PBS), the fluorescence was measured at an excitation/emission wavelength at 485 nm/535 nm, respectively. Mitochondrial O_2_^• ‒^ was detected in adipocytes with a specific fluorescent dye, Mitosox Red (Invitrogen, Carlsbad, CA, USA) and measuring fluorescence at an excitation/emission wavelength of 510 nm and 580 nm, respectively. Mitochondrial membrane potential (ΔΨm) was determined using the mitochondria-specific fluorescent dye, JC-1 (Thermo Fisher, Skokie, IL, USA), according to manufacturer’s instructions. JC1 aggregates were detected at 550/590 nm (excitation/emission), while JC1 monomers were detected at 485/535 nm (excitation/emission). The JC1 ratio aggregates/monomers was calculated as an indicator of mitochondrial functionality.

#### 2.5.2. Mitochondrial Content and Activity

In adipocytes, mitochondrial content was evaluated using Mitotracker Red (Mitotracker Deep Red FM, Invitrogen) measuring fluorescence intensity at excitation and emission wavelengths of 644 nm and 665 nm, respectively. Citrate synthase (CS) activity and adenosine triphosphate (ATP) content were measured in cell lysates with commercial kits (Cayman Chemical) following the manufacturer’s protocol. Oxygen consumption rate (OCR) was also measured using a kit according to the manufacturer’s instructions (Abcam, Cambridge, UK).

### 2.6. Anti-Adipogenic Potential

3T3-L1 cells were seeded in 6-well plates and induced to differentiation in the presence of the extracts (31–500 μg/mL) during the differentiation process.

#### 2.6.1. Determination of Cellular Lipid Accumulation

Oil Red O (ORO) lipid staining was carried out as previously described [32]. Lipid staining was performed at day 10–12. ORO stock solution was prepared dissolving 0.35% w/v ORO in isopropanol overnight. Cells were fixed with formalin and a working ORO solution was added to each well. ORO staining was eluted with 100% isopropanol and the absorbance was measured at 500 nm.

#### 2.6.2. Assessment of Lipolysis in Adipocytes

The culture media collected from fully differentiated adipocytes after a 24 h treatment with the extracts (31–500 μg/mL) was tested for glycerol and the cell lysates for TAG and lipase activity as described in Section 2.4.2.

#### 2.6.3. Evaluation of Adipocyte Brown Differentiation

To evaluate adipocyte browning, mature adipocytes differentiated in the presence of extracts were used to measure mitochondrial content, CS activity, and ATP content as described in Section 2.5.2.

### 2.7. Insulin Sensitizing Potential

Insulin resistance was triggered in adipocytes by CM-stimulation, as described in Section 2.4.

#### 2.7.1. Quantification of Glucose Uptake

The 3T3-L1 cells differentiated in a black 96-well plate with clear bottom were treated with CM and extracts (31–500 μg/mL) as indicated. After 24 h treatment, the cells were incubated for 1 h in glucose-free DMEM with 100 μM 2-deoxy-2-((7-nitro-2,1,3-benzoxadiazol-4-yl)amino)-D-glucose. At that moment, the cells were washed with PBS, and the fluorescence was detected at an excitation/emission wavelength of 485 nm/535 nm.

#### 2.7.2. Determination of GLUT4 Translocation

The 3T3-L1 cells were cultured on 8-well chambers for cell culture and immunofluorescence, differentiated for 12 days, and treated with CM for 24 h, as previously described. Cells were cultured overnight in low-glucose (5.5 mM) serum-free DMEM supplemented with 0.25% FBS and treated along with the extracts (31–500 μg/mL); then, cells were starved for 4 h and stimulated with 100 nM insulin for 30 min. Glucose transporter type 4 (GLUT4) translocation was determined, according to Luna-Vital et al. [32]. Cells were fixed with 4% paraformaldehyde aqueous solution (Electron Microscopy Sciences) and permeabilized with 0.1% Triton X-100 in PBS. Cells were blocked with Image-iT FX Signal Enhancer (Life Technologies) and incubated with GLUT4 antibody overnight. Cells were washed with PBS and incubated with Alexa Fluor 568 Goat Anti-Rabbit IgG (Life Technologies) secondary antibody (1:200). Cells were cured with ProLong Gold antifade reagent with DAPI (Life Technologies). The microscopy chamber plate was stored at 4 °C in the dark until analysis. Samples were imaged using a 63 × /1.4 Oil DIC M27 objective with a Zeiss LSM 880 laser-scanning confocal microscope (Carl Zeiss AG, Germany). To evaluate GLUT4 translocation to the membrane, the fluorescence intensity, or GLUT4 expression, was measured in the membrane of the cells and normalized using the same laser gain (650) for all the samples. For the total GLUT4 measurement, the intensity of the whole picture was taken in consideration and normalized to the nuclear DAPI staining.

#### 2.7.3. Evaluation of Insulin Signaling Pathway Phosphorylation Pattern

After the 24 h treatments incubation, the cells were starved and stimulated with 10 ng/mL insulin for an extra 30 min. Cell lysates were applied to each one of the array slides following manufacturer instructions. An antibody array, including proteins from insulin, PI3K/AKT, AMPK, MAPK, NF-κB signaling pathways, was used following the manufacturer instructions (Insulin Receptor Phospho Antibody Array, Full Moon BioSystems^®^, Sunnyvale, CA, USA). Results were analyzed as previously described [32]. The arrays were labeled with a Cy3-labeled streptavidin solution (0.5 mg/mL) (Sigma Aldrich, S6402). The slides were scanned on a GenePix4000B scanner and the images were analyzed with GenePix Pro 6.0 (Molecular Devices, Sunnivale, CA). Fluorescence signal of each array spot was quantified; the mean values and the standard deviations of the replicates were calculated.

### 2.8. Bioinformatic Analysis

Functional enrichment analysis was performed using IPA v9.0 (Ingenuity^®^ Systems). Downstream effect analysis was performed to identify biological functions that are expected to be increased/decreased from the observed protein phosphorylation pattern in each sample; significant predictions were considered when biological functions presented an absolute z-score of ≥ 2 (positive indicate activation, negative indicate inhibition). The differentially phosphorylated proteins were categorized based on the biological process and analyzed for Kyoto Encyclopedia of Genes and Genomes (KEGG) pathway enrichment analysis by using the KEGG database.

### 2.9. Statistical Analysis

**Figure 1:**
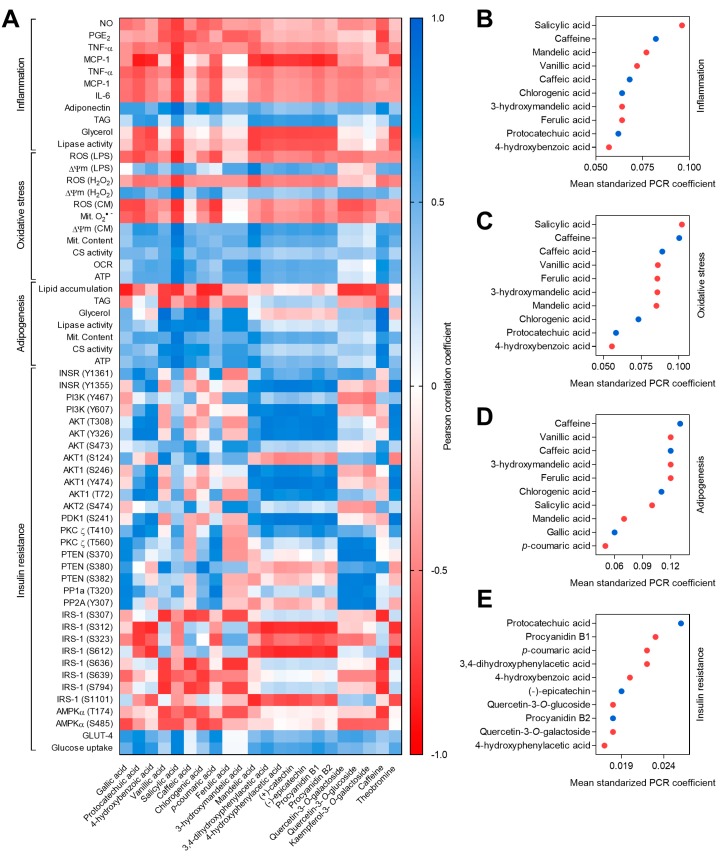
Heat map of the Pearson correlation coefficient established among observed in vitro effects and the concentration of different phytochemicals in coffee and cocoa by-products (**A**) and the top ten mean standardized principal component regression (PCR) coefficients of the regression constructed among the phytochemicals identified in coffee husk, coffee silverskin, and cocoa shell aqueous extracts and the potential of the extracts in the different biomarkers of inflammation (**B**), oxidative stress (**C**), adipogenesis (**D**), and insulin resistance (**E**). Blue (●) and red (●) circles indicate major (one of the ten in higher concentration) and minor phytochemicals, respectively.

Samples were prepared in triplicate, and determinations were performed in triplicate. Results are presented as the mean ± standard deviation (SD) (*n* = 3) and were analyzed by one-way analysis of variance (ANOVA) and post hoc Tukey test. Differences were considered significant at *p* < 0.05 [33,34,35]. The statistical analysis of the results was performed using SPSS 23.0. Non-linear regressions and half maximal effective concentration (*EC*_50_), and 30% effective concentration (*EC*_30_) values were calculated using GraphPad Prism 7. Bi- and multivariate analyses, including Pearson correlations and principal component regression (PCR) were performed with XLSTAT 2018 for Microsoft Excel 2016. All the results were represented using GraphPad Prism 7.0. Pearson correlation. PCR analysis were carried out using the concentration of each one of the compounds in the cell media after coffee husk, coffee silverskin, and cocoa shell extracts application, as well as the values for each of the biomarkers of inflammation, oxidative stress, adipogenesis, and insulin resistance at the respective concentration. A non-treated cells control was also included and a concentration equal to zero for each of the phytochemicals was assigned. PCR analysis is a regression method in two steps; first, a principal component analysis (PCA) is carried out in the *x* variables (concentration of phytochemical) to reduce the dimensionality of the data extracting the factors with the higher variation; the second step is to do a linear regression between the sample scores on the most significant factors in *x* and *y* axes of the PCA. This method reduced collinearity between the predictors (phytochemicals concentration), since in a multiple linear regression model highly intercorrelated predictors may lead to an overfit model with inflated regression coefficients and *p*-values. PCR method takes care of the multicollinearity by using the principal components as new predictor variables to explain the observed variability without considering the response variable. Only significant (*p* < 0.05) Pearson correlation and PCR coefficients were discussed even if all the results were depicted as a heat map (Figure 1A and Figure S2).

## 3. Results and Discussion

### 3.1. Coffee By-Products were Mainly Composed of Chlorogenic Acid and Caffeine whereas Cocoa Shell Primarily Contained Methylxanthines

The phytochemical composition of coffee and cocoa by-products, namely coffee silverskin, coffee husk, and cocoa shell was studied (Table 1).

A total of 20 phenolic compounds and two methylxanthines were identified among the three by-products. The composition of the three by-products was different. Coffee husk and silverskin shared 10 compounds, whereas a total of eight of their phytochemicals was identified in cocoa shell. Coffee husk was mainly composed of caffeine and chlorogenic acid, also containing a significant concentration of protocatechuic acid and flavonols, mainly kaempferol-3-*O*-galactoside. Previous studies also reported similar concentrations of these compounds in coffee husk, as well as the presence of rutin [36]. Coffee silverskin presented a significantly higher (*p* < 0.05) concentration of caffeine (19.2 mg/g) and a lower concentration of chlorogenic acid. This coffee by-product has been one of the most characterized by-products, Bresciani et al. [37] demonstrated the presence of 10 different chlorogenic acids, the 3- and 5-caffeoylquinic acids being the most abundant ones. On the other hand, cocoa shell was primarily composed of theobromine and caffeine, the main phenolics being protocatechuic acid and flavan-3-ols, both monomers ((+)-catechin and (−)-epicatechin), and dimers (procyanidin B2). Other authors have recently reported a similar concentration of theobromine in hydroalcoholic acidic aqueous extracts [38]. Additionally, these abovementioned polyphenols were previously shown to have different reduction potentials, thereby exhibiting various properties in modulation of redox signaling pathways [39,40]. Given these results, it was expected that coffee and cocoa by-products, with different phytochemical composition, would present different biological activities.

### 3.2. Caffeine and Phenolics in Coffee and Cocoa By-Products Reduced Inflammation in Macrophages and Adipocytes

The aqueous extracts of coffee and cocoa by-products did not exert cytotoxicity in macrophages nor pre-adipocytes or mature adipocytes at the tested doses (31–500 µg/mL) (Figure S1). Extracts significantly reduced NO production (23.0–77.5%) and PGE_2_ (14.1–70.7%) in LPS-stimulated macrophages (Table S1). NO and PGE_2_ play a key role in the development of inflammatory diseases involved in the immune response produced by cytokine-activated macrophages [41,42]. Besides, TNF-α (14.1–50.1%) and MCP-1 (8.0–99.4%) release were reduced by coffee and cocoa by-product extracts. TNF-α is considered the main inflammatory stimuli in the adipose tissue while MCP-1 is involved in macrophage recruitment in adipose tissue [43]. Together, they are implicated in the establishment of a paracrine inflammatory interaction between macrophages and adipocytes in obese conditions [44]. As observed (Table 2), coffee husk exhibited the lowest *EC*_30/50_ for the secretion of NO, while coffee silverskin and cocoa shell did for PGE_2_ and TNF-α and MCP-1, respectively.

These effects were mainly correlated with the concentration of salicylic acid (*r* > 0.72, *p* < 0.001) (Figure 1). In previous studies, salicylic acid has been reported as an anti-inflammatory agent [45,46]; this well-recognized anti-inflammatory compound could reduce TNF-α and IL-6 in RAW264.7 and THP-1 macrophages. Moreover, salicylic acid is the active bioavailable form of the well-known aspirin (acetylsalicylic acid), a common medication used to treat inflammation [47,48].

Principal component regressions (PCR) were calculated to obtain a less biased quantitative value (standardized PCR coefficients) of the effect of phytochemicals on the observed bioactivities (Figure S2). Thus, we found an association of caffeine with NO, PGE2, and TNF-α release, and correlation of caffeine and protocatechuic acid with MCP-1 production. Chlorogenic and caffeic acid also showed a high contribution to the anti-inflammatory effects of coffee and cocoa by-products extracts. Caffeine previously demonstrated anti-inflammatory effects in LPS-stimulated RAW264.7 macrophages, reducing inducible nitric oxide synthase (iNOS), NO, cyclooxygenase-2 (COX-2), and IL-6 via NF-κB and p38 MAPK pathway inhibition [49]. Chlorogenic and caffeic acids have also evidenced anti-inflammatory potential in LPS-stimulated RAW264.7 macrophages decreasing iNOS, NO, COX-2, PGE2, and inflammatory cytokines, including IL-6, TNF-α, and MCP-1 [28,50].

Along with obesity development, the progression of adipocyte hypertrophy and adipose tissue dysfunction evokes an exacerbated release of fatty acids, hormones, and pro-inflammatory cytokines [51]. Here we observed that in 3T3-L1 adipocytes, the extracts abrogated the secretion of TNF-α (8.0–66.7%), MCP-1 (37.1–71.2), IL-6 (21.0–40.1), and restored the production of adiponectin (1.0–75.5%). Additionally, they diminished inflammation-derived lipolysis, maintaining the content of TAG (23.3–134.3%), and reducing glycerol release (7.1–99.9%) through the inhibition of lipases activity (8.9–99.7%). Coffee husk presented the lowest *EC*_30/50_ values for TNF-α, MCP-1, and IL-6, whereas cocoa shell was the most potent inhibition inflammation-derived lipolysis (Table 2). Salicylic and protocatechuic acids also correlated with the secretion of adipokines; caffeine correlated with adiponectin production (*r* = 0.727, *p* < 0.01), and lipolysis markers were better associated with protocatechuic acid. PCR coefficients demonstrated the contribution of *p*-coumaric in diminishing MCP-1 and IL-6 releases. Chlorogenic acid exhibited a high contribution to the promotion of adiponectin release. Salicylic acid has been shown to decrease the inflammatory interplay of macrophages–adipocytes in CM-treated 3T3-L1 adipocytes [48]. It reduced TNF-α and IL-6 secretion and promoted adiponectin release. Furthermore, protocatechuic acid has shown to decrease TNF-α, MCP-1, and IL-6 in the adipose tissue both in vitro (3T3-L1 cells) and in vivo (humans) [27,52] Meanwhile, *p*-coumaric acid have exhibited anti-inflammatory properties in RAW264.7 cells suppressing COX-2, iNOS, and TNF-α expression via inhibition of NF-κB and p-38 MAPK pathways [53].

The top ten phytochemicals analyzed contributing to the anti-inflammatory properties of coffee and cocoa by-products are shown in Figure 1B. Caffeine, chlorogenic, and caffeic acids were the 3 major compounds showing anti-inflammatory potential, whereas salicylic, mandelic, and vanillic acids were the minor compounds with the highest contribution.

### 3.3. Phenolic Compounds Reduced Oxidative Stress in Macrophages and Adipocytes and Caffeine Preserved Adipocyte Mitochondrial Function

Obesity has been linked not only to metabolic dysfunction but also to oxidative stress. This stress derives from inflammation, as part of the immune response of macrophages, and from mitochondrial dysfunction, due to the excess of substrates to be oxidized [54]. Thus, together with the inflammatory cascade triggered by LPS-stimulation, an increase of oxidative state, and a deficient mitochondrial function were observed in macrophages. Coffee and cocoa by-products aqueous extracts significantly (*p* < 0.05) scavenged ROS produced by both LPS (36.0–99.5%) and H_2_O_2_ (30.8–86.9%) stimuli (Table S2), maintaining the ΔΨm. Cocoa shell exhibited the highest antioxidant capacity (lowest values of *EC*_30/50_) (Table 3). LPS-derived ROS was correlated with protocatechuic acid concentration (*r* = −0.710, *p* < 0.01) as well as H_2_O_2_-derived ROS (*r* = −0.571, *p* < 0.05). The recovery of ΔΨm upon both stimuli was significantly associated with mandelic and *p*-coumaric acid concentration (*r* = 0.802, *p* < 0.001 and *r* = 0.644, *p* < 0.01, respectively). PCR coefficient denoted the contribution of caffeic and chlorogenic acids on the prevention of ΔΨm loss with LPS and H_2_O_2_ treatments, respectively. Furthermore, in adipocytes, the extracts reduced ROS (21.5–66.4%) and particularly mitochondrial O_2_^• ‒^ (18.1–52.6%). The loss of ΔΨm was arrested by 11.0–96.6%. Coffee husk showed the lowest *EC*_30/50_ values for ROS production in adipocytes (Table 3).

ROS inhibition was correlated with protocatechuic and *p*-coumaric acids (*r* = −0.716, *p* < 0.01 and *r* = −0.779, *p* < 0.001, respectively). Mitochondrial O_2_^• ‒^ followed the same behavior. ΔΨm was correlated with the concentration of mandelic acids (*r* = −0.787, *p* < 0.001). Moreover, PCR showed a high contribution of *p*-coumaric and gallic acid in the attenuation of ROS and O_2_^• ‒^ in adipocytes, whereas ΔΨm was associated with the concentration of caffeine and caffeic acid. Protocatechuic acid has evidenced its potent antioxidant properties in vitro and in vivo [55]. In macrophages, protocatechuic acid up-regulated enzymatic antioxidant defenses through JNK-mediated Nrf2 activation [56]. Likewise, caffeic acid previously has exhibited protective effects against mitochondrial oxidative stress, scavenging H_2_O_2_ [57], and preserving glutathione levels upon *tert*-butyl hydroperoxide-induced oxidative stress in HepG2 cells [58]. Additionally, caffeine has demonstrated to possess ROS scavenging properties and capacity to maintain ΔΨm [59].

The loss of mitochondrial function derived from an inflammatory and oxidative environment has been associated with increased insulin resistance in the adipose tissue [60]. Here, treating adipocytes with coffee and cocoa by-products preserved mitochondrial content (12.1–144.8%) and activity, measured as CS activity (75.6–150.8%), OCR (0.6–66.5%), and the production of ATP (11.4–108.3%) (Table S2). Coffee silverskin was the significantly (*p* < 0.05) most potent treatment in the conservation of mitochondrial content and activity, exhibiting *EC*_30/50_ values lower than 31 µg/mL except for OCR (125 µg/mL) (Table 3). These effects were associated with the concentration of caffeine (*r* ≥ 0.563, *p* < 0.05). Protocatechuic acid was linked to the protection of mitochondrial content (*r* = 0.571, *p* < 0.05); caffeic acid was associated with OCR (*r* = 0.626, *p* < 0.01); and mandelic acid with OCR and ATP content (*r* ≥ 0.746, *p* < 0.01). PCR highlighted the contribution of caffeic acid and caffeine in the preservation of mitochondrial content and activity (CS, activity, OCR, and ATP content). Protocatechuic acid has proved potential on mitochondrial function in vitro and in vivo, preventing derived cell apoptosis [61,62]. Caffeic acid has displayed protective effects on mitochondrial function under oxidative stress conditions via activation of *Nrf2* [58]. Furthermore, the effects of caffeine have been investigated; this methylxanthine exhibited the ability to increase OCR and ATP production in mice brain [59].

The top ten compounds analyzed ordered by contribution to the preventive properties of coffee and cocoa by-products against oxidative stress and mitochondrial dysfunction are shown in Figure 1C. Again, caffeine, and chlorogenic and caffeic acids were the three compounds identified in a higher concentration that exhibited the best antioxidant properties. On the other hand, salicylic, vanillic, and ferulic acids were the minor compounds with the highest contribution.

### 3.4. Caffeine and Phenolics in Coffee and Cocoa By-Products Attenuated Adipogenesis and Promoted Adipocyte Browning

Besides favoring inflammatory and oxidative processes, the adipose tissue continues expanding in energy imbalance conditions (high caloric diets and reduced physical activity). Reducing lipid accumulation and promoting lipolysis in the first stages of adipose tissue expansion can be considered to be anti-adipogenic strategies [63]. Extracts from coffee and cocoa by-products reduced lipid accumulation (4.1–44.5%) (Table S3). The lowest (*p* < 0.05) *EC*_30/50_ value was that of coffee husk (Table 4). This lipid-reducing effect may be either owing to cell proliferation or the inhibition of the preadipocyte differentiation (adipogenesis) [63]. This effect mainly correlated with the concentration of gallic, chlorogenic, and *p*-coumaric acids (*r* = −0.870, −0.843, and −0.843, respectively, *p* < 0.0001). PCR analysis supported those results. Gallic acid proved to have anti-adipogenic activity in 3T3-L1 adipocytes by inhibiting *Pparγ*, *Scd-1*, and *Fasn* expression [64]. Besides exerting these effects, *p*-coumaric acid was able to down-regulate the expression of the transcription factor *CEBPα*, *Lpl*, *AdipoQ*, and *Fabp4*. Additionally, chlorogenic acid reduced lipid accumulation via *Plin1* and *Srebp1* down-regulation. The impact of the coffee and cocoa by-products extracts on lipogenesis/lipolysis in mature adipocytes was further assessed, and the results are shown in Table 4 and Table S3. Extracts reduced TAG content by 11.9–63.0% via lipolysis stimulation, enhancing glycerol release (0.4 to 3.2-fold) and lipase activity (6.5–45.6%). Coffee silverskin was found to be the most effective treatment exhibiting the lowest *EC*_30/50_ values. The effect in glycerol significantly correlated with the content of vanillic and chlorogenic acids (*r* = 0.908 and 0.845, respectively, *p* < 0.0001) and the concentration of caffeine (*r* = 0.899, *p* < 0.0001); similarly, these compounds correlated with the activation of lipases activity. Both the Pearson correlation and PCR analysis revealed the contribution of caffeic acid. Vanillic acid presented a strong effect reducing TAG content in 3T3-L1 adipocytes through the reduction of *AdipoQ*, *Plin1*, and *Fabp4* expression [64]. Chlorogenic acid has shown to increase lipolysis and up-regulate the expression of lipases (*Hsl* and *Dgat1*) [65,66]. Similarly, caffeine has exhibited potential to up-regulate HSL and AGTL and increase lipolysis, reducing TAG content in 3T3-L1 cells [67,68].

Furthermore, it has been shown that during differentiation, adipocytes can acquire a brown-like or beige phenotype, a process known as adipocyte browning. These beige adipocytes exhibit higher mitochondrial content and activity [69,70]. Thus, coffee and cocoa by-products increased (1.0–26.6%) the mitochondrial content as well as increased mitochondrial citrate synthase activity by 9.0–54.5%; the production of ATP was also increased (2.9–48.0%). The lowest *EC*_30/50_ values were shown for coffee silverskin treatment (Table 4). The increase in mitochondrial content was associated with the concentration of salicylic and mandelic acids in the extracts (*r* = 0.831 and 0.735, respectively, *p* < 0.001). However, the promotion of mitochondrial CS activity and the production of ATP better correlated with the content of salicylic acid and caffeine (*r* = 0.789 and 0.774, respectively, *p* < 0.001). Nonetheless, the contribution of caffeic acid, beyond caffeine, was demonstrated by PCR analysis. The browning effects of salicylic acid has been associated with its capacity to increase *Ucp1*, *Pparα*, and *Elovl3* expression [71]. The effects of caffeine on adipocyte browning have been intensely studied. Caffeine prompts mitochondrial biogenesis and UCP1 expression, increasing the oxidative capacity of mitochondria and ATP production, via PGC-1α pathway activation, and evidencing enhanced thermogenesis in humans [72,73]. Likewise, caffeic acid has been suggested to be able to increase mitochondrial content and prompt its oxidative activity in adipocytes [28]. The use of nutritional interventions, natural products, and phytochemicals to stimulate mitochondrial biogenesis and bioenergetics and attenuate oxidative stress are current strategies for the prevention and treatments of chronic metabolic disorders [74].

The ten compounds with the highest potential to inhibit adipogenesis and elicit adipocyte browning are described in Figure 1D. Caffeine, chlorogenic, and caffeic acids were the main contributor among those phytochemicals identified in higher concentration in coffee and cocoa by-products. Vanillic, 3-hydroxymandelic, and ferulic acids were the minor compounds exhibiting the highest contribution.

### 3.5. Phenolic Compounds Counteracted Insulin Resistance through the Modulation of Insulin Signaling and the Promotion of GLUT4 Translocation

**Figure 2:**
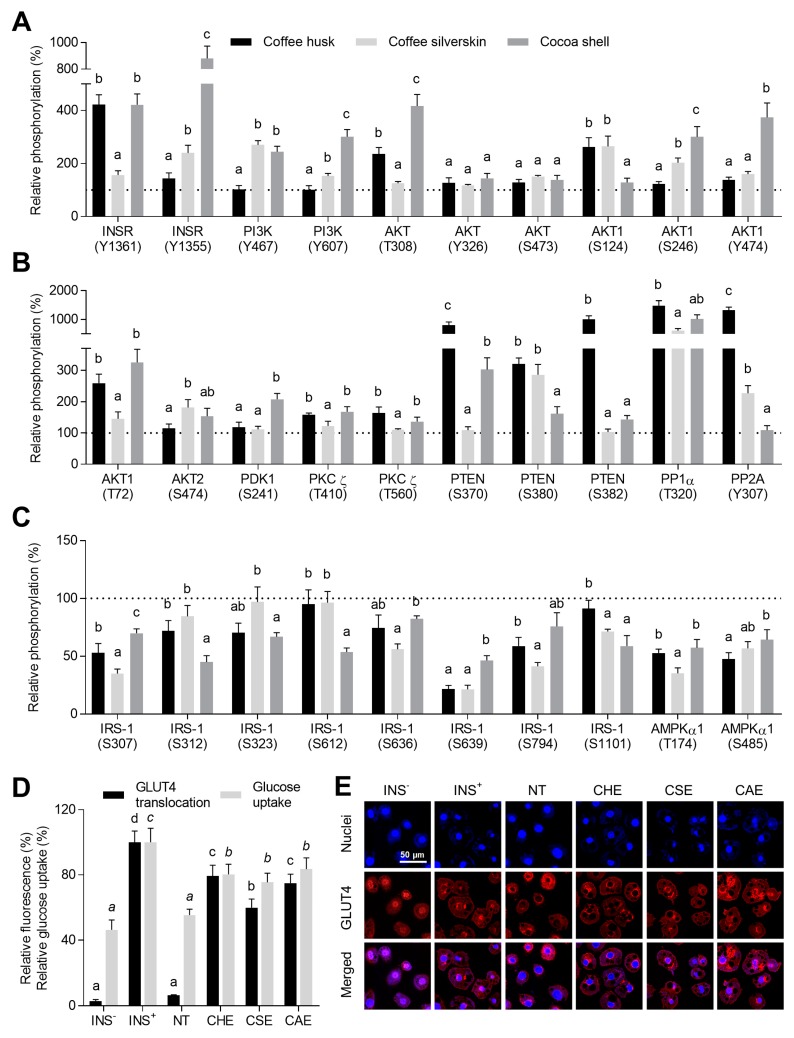
Insulin-sensitizing effects of coffee husk, coffee silverskin, and cocoa shell aqueous extracts (125 μg/mL) on adipocyte treated with the conditioned media from macrophages. Up-phosphorylated proteins (**A**,**B**) and down-phosphorylated proteins (**C**) from the insulin signaling pathway. GLUT4 translocation into the cell membrane measured as the relative fluorescence intensity between GLUT4 and the nucleus and glucose uptake (**D**), and confocal laser scanning microscopy representative images (**E**). INS: insulin; NT: non-treated cells; CHE: coffee husk extract; CSE: coffee silverskin extract; CAE: cocoa shell extract.

Upon insulin binding with its receptor, a sequence of reactions conduces to GLUT4 translocation into the membrane and the subsequent glucose uptake. In obese conditions, inflammation derives in insulin resistance, which is associated with the development of type 2 diabetes [75,76]. The extracts from coffee and cocoa by-products counteracted the inhibition of insulin signal transduction via modulation of the phosphorylation of key proteins in the insulin pathway (Figure 2). From a total of 30 protein involved in the insulin receptor signaling pathway, 20 protein were up-phosphorylated and 10 down-phosphorylated. CHE and CAE promoted INSR tyrosine phosphorylation. CSE and CAE potentiated PI3K phosphorylation (52.8–201.2%). That resulted in augmented AKT phosphorylation (from 16.9 to 316.5%). Lately, extracts prompted PKC ζ phosphorylation (9.9–67.8%), which would result in GLUT 4 translocation into the cell membrane [77]. Furthermore, extracts inhibit the action of diverse phosphatases (PTEN, PP1α, and PP2A) through their phosphorylation (from 1.4 to 14.8-fold). IRS-1 was down-phosphorylated by at least one of the extracts in six different serine residues, which promoted insulin signaling. As a result, GLUT4 was significantly (*p* < 0.05) more translocated to the membrane (59.8–79.3%) and glucose uptake stimulated (75.4–80.4%) in comparison to the non-treated control. INRS phosphorylation was associated with the content of protocatechuic acid and theobromine (*r* = 0.763 and 0.833, respectively, *p* < 0.001). PCR denoted the contribution of (+)-catechin. Likewise, the phosphorylation of PI3K correlated with the concentration of (+)-catechin and theobromine (*r* = 0.818 and 0.817, respectively, *p* < 0.001). AKT phosphorylation, in its different residues, correlated with protocatechuic acid and procyanidin B1 (T308), theobromine and caffeine (S326 and S473, respectively), chlorogenic acid and caffeine (S124), theobromine (S246), (+)-catechin, (−)-epicatechin, procyanidin B2 (Y474), and protocatechuic acid (T72). PCR exhibited a significant contribution in the phosphorylation of AKT of protocatechuic acid and procyanidin B1 (Y326) and corroborated the results obtained using Pearson correlations. PKC ζ phosphorylation was associated with protocatechuic and *p*-coumaric acids (T410 and T560). PTEN exhibited an association with chlorogenic (S380), gallic acid, and kaempferol-3-*O*-galactoside (S382). Similar behavior demonstrated PP1α and PP2A. Moreover, the results from PCR analysis gave similar information on the contribution of those phytochemicals. The down-phosphorylation of IRS-1 mainly correlated with the concentration of protocatechuic and caffeic acids, caffeine, and theobromine. GLUT4 translocation correlated with the concentration of protocatechuic and gallic acids (*r* = 0.699 and 0.642, respectively, *p* < 0.01). Glucose uptake was associated with the concentration of protocatechuic acid (*r* = 0.746, *p* < 0.001). The contribution of *p*-coumaric acid was also significant, as observed in both the Pearson correlation and PCR coefficients.

Protocatechuic acid possesses insulin-mimicking both in vitro and in vivo. Engaging the INSR and including a reversion of INS-1 serine phosphorylation, and activation of insulin/PI3K/AKT and AMPK signaling pathways, protocatechuic acid triggers a positive regulation of and glucose uptake through GLUT4 translocation [78]. Likewise, gallic acid induces GLUT4 translocation and glucose uptake activity in 3T3-L1 cells via PKC ζ activation [79]. Insulin resistance is strongly associated with the pathogenesis of type 2 diabetes and obesity and results from a sustained low-grade inflammatory status and the loss of the mitochondrial function [80]. Hence, the alleviation of these conditions and the modulation of insulin sensitivity are needed in the prevention of the development and exacerbation of obesity and diabetes outcomes.

The top ten phytochemicals analyzed from coffee and cocoa by-products with the highest potential to prevent insulin resistance development are included in Figure 1E. Protocatechuic acid, (−)-epicatechin, and procyanidin B2 were the major compounds contributing to these effects. Procyanidin B1, *p*-coumaric and 3,4-dihydroxyphenilacetic acids, in turn, the minor compounds exhibiting the highest contribution.

### 3.6. Phytochemicals from Coffee and Cocoa By-Products Regulated Protein Phosphorylation thereby Preventing Inflammation, Oxidative Stress, and Insulin Resistance

**Figure 3:**
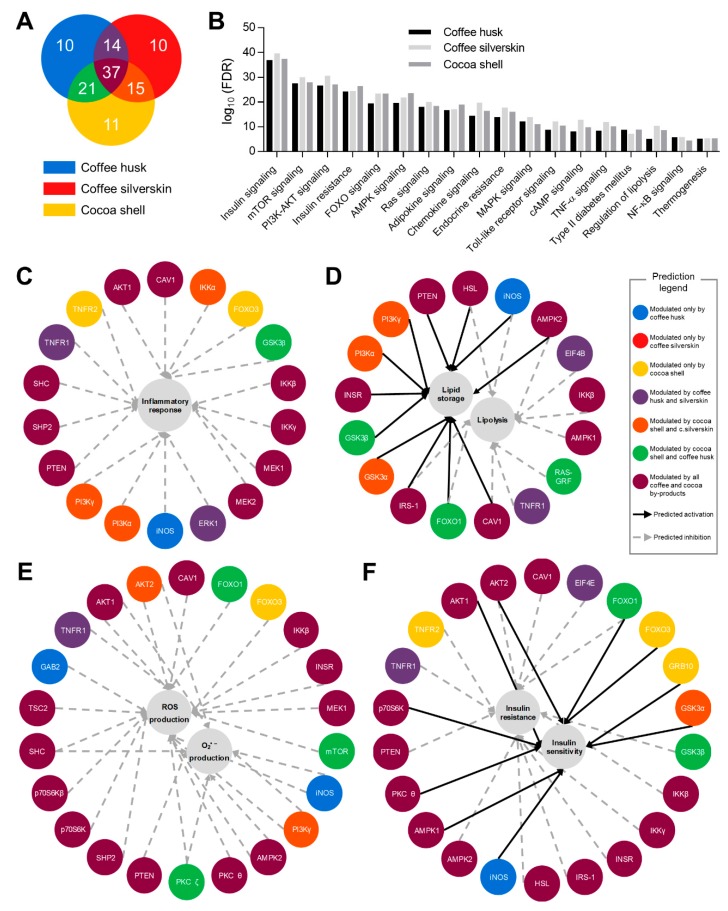
Venn diagram showing the overlap of differentially phosphorylated proteins in adipocytes upon the treatment with coffee husk, coffee silverskin, and cocoa shell aqueous extracts (31–500 μg/mL) (**A**). KEGG pathways associated with the differentially phosphorylated proteins (**B**). Downstream effect analysis of coffee and cocoa extracts on inflammatory response (**C**), lipid storage and lipolysis (**D**), oxidative stress (**E**), and insulin resistance (**F**).

Beyond modifying the phosphorylation of proteins in the insulin receptor pathway, coffee and cocoa by-product aqueous extracts modulated the phosphorylation state of proteins associated with pathways related to inflammation and oxidative stress (Figure 3). Coffee husk significantly (*p* < 0.05) modulated the phosphorylation of 82 out of 122 (67%) phosphorylation sites evaluated, whereas coffee silverskin modified 76 and cocoa shell 84 (62 and 69%, respectively) (Figure 3A; Table S3). Among the three treatments, the phosphorylation pattern was different. They shared 51–58 phosphorylation sites when comparing among pairs, and only 37 phosphorylation sites were modulated by all the treatments. Among the top pathways accounting with modified proteins, the most significant ones were insulin, mTOR, PI3K-AKT, FOXO, and AMPK signaling pathways (Figure 3B).

Downstream effect analysis validated the effects analyzed in adipocytes for inflammation, oxidative stress, and insulin resistance (Figure 3C–F). The inflammatory response in adipocytes was mediated by the phosphorylation of proteins from the TNF-α (TNFR1, TNFR 2), NF-κB (IKKβ, IKKγ), and MAPK (MEK1, MEK2, ERK1) pathways, among others (Figure 3C). Lipid accumulation and lipolysis in inflammatory conditions were regulated by the modulation of the phosphorylation of proteins related to the insulin (INSR, IRS-1, PI3K, AKT, FOXO) and the TNF-α (TNFR, IKKβ, iNOS) signaling pathways and HSL (Figure 3D). Inflammation in the adipose tissue causes lipolysis and the release of free fatty acids, serving as ligands for toll-like receptor-4 (TLR4) and further activating NF-κB and JNK, leading mediators of inflammation [81]. Thus, the reduction of inflammation in adipocytes results in lower lipolysis (higher accumulation of lipid) through the maintaining of insulin sensitivity. As observed in Figure 2B, the main compounds identified in coffee and cocoa by-products responsible for the anti-inflammatory properties were caffeine, caffeic, chlorogenic, and protocatechuic acids.

Furthermore, the phosphorylation of proteins from insulin (INSR, PI3K, AKT), PKC (PKC ζ, PKC θ), and mTOR (mTOR, p70S6K) pathways was predicted to be affecting the production of ROS and specifically the production of mitochondrial superoxide (Figure 3E). The main phytochemicals contributing to these effects were also caffeine, caffeic, chlorogenic, and protocatechuic acids. It has been reported that mTOR, a downstream of the insulin/PI3K/AKT pathway, depending upon PKC, can be conducive to defective ROS production [82]. Most of these proteins were modulated by both coffee and cocoa by-products. Therefore, the production of ROS and mitochondrial superoxide was repressed.

On the other hand, the most important compounds contributing to the anti-adipogenic properties of coffee and cocoa by-products were caffeine, and chlorogenic and gallic acids. Finally, the modification of the phosphorylation state of proteins in insulin (INSR, IRS-1, AKT), AMPK (PKA), TNF-α (TNFR1, TNFR 2), and NF-κB (IKKβ, IKKγ) signaling pathways triggered an increase in insulin sensitivity in adipocytes in inflammatory conditions (Figure 3F). Among the pro-inflammatory stimuli secreted by macrophages, TNF-α presence proved evidence of conducting insulin resistance through the IRS-1 serine phosphorylation, and inactivation, via activation of NF-κB, ERK, JNK, mTOR, and PKC θ [83]. We might attribute these effects to protocatechuic acid, (−)-epicatechin, and procyanidin B2.

Hence, coffee husk, coffee silverskin, and cocoa shell mediated alleviation of inflammatory processes could also reverse inflammation-triggered oxidative stress, mitochondrial dysfunction, and insulin resistance.

**Figure 4:**
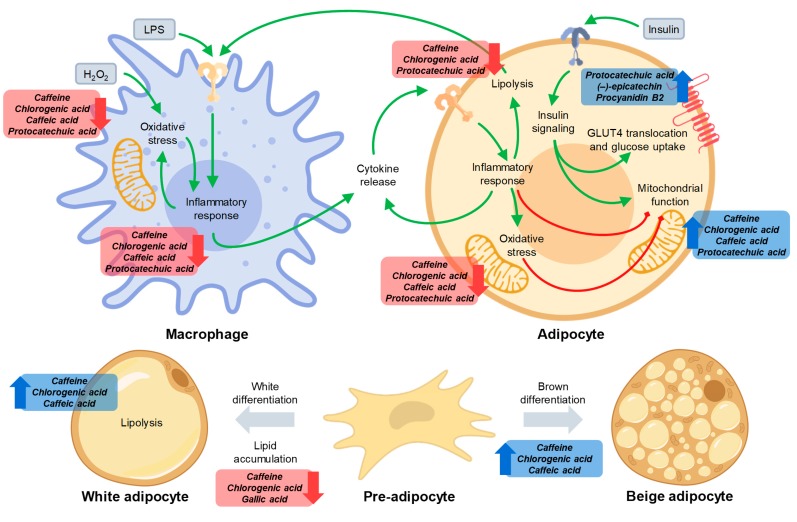
Integrative diagram illustrating the underlying molecular mechanism of the phytochemicals from coffee husk, coffee silverskin, and cocoa shell in inflammation, oxidative stress, adipogenesis, and insulin resistance in macrophages and adipocytes.

A diagram illustrating the mechanism and main actors of the effects in macrophages and adipocytes is shown in Figure 4. As described, the phytochemicals exerting most of the biological effects associated with inflammation, oxidative stress, adipogenesis, and insulin resistance were caffeine, chlorogenic, and protocatechuic acids.

In this comparative study, we have observed that the differential composition of coffee and cocoa by-products is associated with their in vitro biological properties. Both major and minor components of coffee husk, coffee silverskin, and cocoa shell presented a relationship with the potential against inflammation, oxidative stress, adipogenesis, and insulin resistance. Comparing the effects of coffee husk, coffee silverskin, and cocoa shell, we observed (Figure S3) that the best extract for preventing inflammation was that of cocoa shell. Results suggest that oxidative stress was prevented by cocoa shell and coffee silverskin, whereas the anti-adipogenic potential was stronger for coffee silverskin. The ability to counteract insulin resistance was higher for coffee husk. Despite the potential biological activity of phytochemicals identified in coffee and cocoa by-products, phenolic compound bioefficacy is conditioned by their low bioavailability. These phytochemicals are only partially absorbed in the gastrointestinal tract. After being metabolized by the microbiota or in the liver (methylation, sulfation, and glucuronidation), they reach target tissues at low concentrations [71,72]. Future in vivo studies with coffee and cocoa by-products will allow us to ascertain their pharmacokinetic behavior and their efficacy in delaying the development of metabolic disorders related to inflammation and oxidative stress.

## 4. Conclusions

The use of coffee and cocoa by-products as a source of phytochemicals is proposed as a strategy in the prevention of chronic diseases related to inflammation and oxidative stress, such as obesity and diabetes. This study presents an evaluation of the chemical composition of coffee husk, coffee silverskin, and cocoa shell in the inhibition of inflammation, oxidative stress, adipogenesis, and insulin resistance using RAW264.7 macrophages and 3T3-L1 adipocytes. Taken together, our results support the notion that phenolic compounds and methylxanthines from coffee and cocoa by-products can trigger an attenuation of inflammation and oxidative stress in macrophages and adipocytes, counteracting the consequent loss of mitochondrial function, as well as reduce adipogenesis and induce browning in adipocytes, and potentiating insulin sensitivity. Furthermore, we provide new knowledge on the underlying mechanism of action and contribution of the phytochemicals composing coffee husk, coffee silverskin, and cocoa shell. This research revalorizes by-products from coffee and cocoa, therefore, increasing their potential utilization and their conversion into food ingredients, with the added-value potential to prevent chronic metabolic diseases. Additionally, in the future, in vivo studies will be accomplished to validate the safety and efficacy of coffee and cocoa by-products as new ingredients. In conclusion, we established, for the first time, the relationship of the composition of different phytochemicals among coffee and cocoa by-products and their in vitro potential to reduce markers of inflammation, oxidative stress, adipogenesis, and insulin resistance.

## Figures and Tables

**Table 1:** Phytochemical composition of coffee husk, coffee silverskin, and cocoa shell aqueous extracts characterized by UPLC–ESI–MS/MS. Values are expressed as mean ± SD (n = 3). Different letters among rows indicate significant differences (*p* < 0.05) according to ANOVA and Tukey’s multiple range test.

Compound	Concentration (µg/g Extract)			Chemical Structure
	Coffee Husk	Coffee Silverskin	Cocoa Shell	
Hydroxybenzoic acids				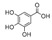
Gallic acid	87.0 ± 5.5^c^	16.9 ± 1.2^a^	19.2 ± 0.4^b^	
Protocatechuic acid	488.4 ± 26.2^b^	44.1 ± 3.4^a^	761.5 ± 47.6^c^	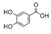
4-hydroxybenzoic acid	13.4 ± 1.3^b^	3.4 ± 0.3^a^	70.1 ± 9.3^c^	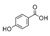
Vanillic acid	22.90 ± 9.62^a^	29.6 ± 0.6^a^	N.D.	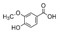
Salicylic acid	3.1 ± 0.1^b^	2.3 ± 0.2^a^	3.3 ± 0.2^b^	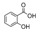
Hydroxycinnamic acids				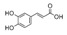
Caffeic acid	57.9 ± 2.0^b^	538.0 ± 54.3^c^	1.9 ± 0.2^a^	
Chlorogenic acid	3456.8 ± 70.6^b^	2791.7 ± 97.3^a^	N.D.	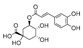
*p*-coumaric acid	8.7 ± 0.2^c^	0.9 ± 0.1^a^	4.2 ± 0.6^b^	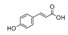
Ferulic acid	N.D.	3.8 ± 0.2	N.D.	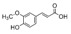
Mandelic acids				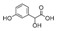
3-hydroxymandelic acid	N.D.	4.4 ± 0.5	N.D.	
Mandelic acid	N.D.	5.1 ± 0.2^a^	11.18 ± 1.2^b^	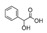
Phenylacetic acids				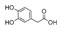
3,4-dihydroxyphenylacetic acid	5.6 ± 2.0^a^	N.D.	25.9 ± 2.4^b^	
4-hydroxyphenylacetic acid	N.D.	N.D.	48.5 ± 4.3	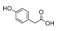
Flavan-3-ols: monomers				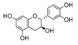
(+)-catechin	1.7 ± 0.2^a^	10.2 ± 1.1^b^	200.8 ± 16.0^c^	
(−)-epicatechin	18.0 ± 2.0^a^	N.D.	222.1 ± 13.8^b^	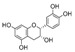
Flavan-3-ols: dimers				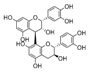
Procyanidin B1	22.3 ± 2.6^a^	N.D.	83.6 ± 7.8^b^	
Procyanidin B2	11.6 ± 1.8^a^	N.D.	219.9 ± 11.4^b^	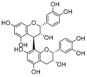
Flavonols				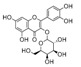
Quercetin-3-*O*-galactoside	54.7 ± 0.5^b^	N.D.	9.3 ± 0.4^a^	
Quercetin-3-*O*-glucoside	57.4 ± 3.7^b^	N.D.	11.12 ± 0.77^a^	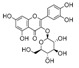
Kaempferol-3-*O*-galactoside	122.6 ± 3.6	N.D.	N.D.	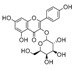
Alkaloids				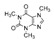
Caffeine	9815.5 ± 15.4^b^	19,219.2 ± 37.6^c^	2433.5 ± 7.8^a^	
Theobromine	N.D.	N.D.	10,035.0 ± 4.5	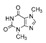

N.D. Non-detected.

**Table 2:** Anti-inflammatory effect of coffee husk, coffee silverskin, and cocoa shell extracts (31–500 μg/mL) regulating NO, PGE_2_, TNF-α, and MCP-1 release in lipopolysaccharide (LPS)-stimulated RAW264.7 macrophages, and TNF-α, MCP-1, IL-6, adiponectin, intracellular triglyceride content, extracellular glycerol release, and lipase activity conditioned media (CM)-stimulated in 3T3-L1 adipocytes. Values are expressed as mean (µg/mL) ± SD (*n* = 3). Different letters among rows indicate significant differences (*p* < 0.05) according to ANOVA and Tukey’s multiple range test.

Biomarkers	Coffee Husk		Coffee Silverskin		Cocoa Shell	
	*EC*_30_	*EC*_50_	*EC*_30_	*EC*_50_	*EC*_30_	*EC*_50_
RAW264.7 macrophages						
NO release	31.5 ± 5.0^a^	73.4 ± 8.9_a_	41.8 ± 4.4^b^	97.5 ± 6.5_b_	40.3 ± 9.8^ab^	94.0 ± 16.5_ab_
PGE_2_ release	134.0 ± 33.7^b^	312.6 ± 48.4_b_	58.0 ± 6.4^a^	135.2 ± 9.4_a_	118.4 ± 13.1^b^	276.4 ± 19.2_b_
TNF-α release	158.8 ± 43.2^a^	370.4 ± 61.7_a_	146.5 ± 29.5^a^	341.8 ± 42.8_a_	106.7 ± 35.7^a^	498.9 ± 50.3_b_
MCP-1 release	87.2 ± 12.1^b^	203.5 ± 17.7_b_	173.9 ± 19.7^c^	420.3 ± 25.0_a_	32.6 ± 4.7^a^	76.1 ± 8.6_a_
3T3-L1 adipocytes						
TNF-α release	59.8 ± 15.3^a^	139.5 ± 21.9_a_	138.0 ± 16.5^c^	321.8 ± 24.1_c_	87.8 ± 10.4^b^	204.9 ± 15.2_b_
MCP-1 release	<31.0	80.0 ± 16.6_a_	<31.0	179.3 ± 36.8_b_	<31.0	132.5 ± 18.8_b_
IL-6 release	88.2 ± 7.6^a^	>500	233.5 ± 86.7^c^	>500	112.5 ± 19.8^b^	>500
Adiponectin release	96.6 ± 9.7^a^	225.4 ± 14.3_a_	95.7 ± 9.8^a^	232.9 ± 16.5_a_	115.2 ± 17.2^a^	268.7 ± 25.1_b_
Triglyceride content	<31.0	60.3 ± 10.0_b_	<31.0	32.6 ± 5.5_a_	<31.0	<31.0
Glycerol release	77.5 ± 30.2^a^	265.7 ± 42.2_c_	68.5 ± 23.4^a^	187.2 ± 25.7_b_	<31.0	26.7 ± 2.8_a_
Lipase activity	107.1 ± 20.5^b^	249.8 ± 29.7_c_	70.5 ± 8.2^a^	148.0 ± 12.3_b_	<31.0	30.2 ± 4.4_a_

**Table 3:** Protective effect of coffee husk, coffee silverskin, and cocoa shell extracts (31–500 μg/mL) against oxidative stress and mitochondrial dysfunction regulating ROS and ΔΨm in RAW264.7 macrophages and ROS, mitochondrial O_2_^• ‒^, ΔΨm, mitochondrial content, citrate synthase (CS) activity, oxygen consumption rate (OCR), and ATP content in 3T3-L1 adipocytes. Values are expressed as mean (µg/mL) ± SD (*n* = 3). Different letters among rows indicate significant differences (*p* < 0.05) according to ANOVA and Tukey’s multiple range test.

Biomarkers	Coffee Husk		Coffee Silverskin		Cocoa Shell	
	*EC*_30_	*EC*_50_	*EC*_30_	*EC*_50_	*EC*_30_	*EC*_50_
RAW264.7 macrophages						
ROS (LPS)	<31.0	48.2 ± 2.4_b_	<31.0	69.5 ± 6.6_c_	<31.0	34.7 ± 2.7_a_
ΔΨm (LPS)	>500	>500	167.7 ± 22.6^a^	391.3 ± 33.0_a_	143.5 ± 36.9^a^	334.9 ± 52.8_a_
ROS (H_2_O_2_)	51.7 ± 9.3	120.7 ± 13.5_c_	<31.0	71.7 ± 3.2_b_	<31.0	46.6 ± 3.5_a_
ΔΨm (H_2_O_2_)	<31.0	33.6 ± 3.7_a_	32.4 ± 3.5^a^	75.6 ± 6.6_b_	41.9 ± 8.6^a^	97.8 ± 12.4_c_
3T3-L1 adipocytes						
ROS	54.8 ± 10.5^a^	128.1 ± 15.2_a_	150.2 ± 39.7^c^	350.6 ± 56.9_b_	82.4 ± 16.1^b^	192.3 ± 23.4_a_
Mitochondrial O_2_^• ‒^	86.4 ± 24.5^a^	201.6 ± 35.0_a_	207.8 ± 57.2^b^	>500	94.8 ± 28.5^a^	221.2 ± 40.4_a_
ΔΨm	140.9 ± 14.8^c^	336.0 ± 12.5_b_	58.0 ± 6.3^a^	135.3 ± 9.2_a_	94.8 ± 18.5^b^	161.5 ± 22.9_a_
Mitochondrial content	40.2 ± 7.6	96.0 ± 8.6_b_	<31.0	43.2 ± 6.8_a_	<31.0	74.9 ± 12.1_b_
CS activity	<31.0	<31.0	<31.0	<31.0	<31.0	<31.0
OCR	399.0 ± 66.1^b^	>500	125.01 ± 23.5^a^	291.8 ± 34.0_a_	158.11 ± 24.6^a^	368.8 ± 35.9_b_
ATP content	80.7 ± 11.3^b^	188.2 ± 16.5_c_	<31.0	59.4 ± 7.6_a_	39.4 ± 8.3^a^	91.9 ± 14.3_b_

**Table 4:** Anti-adipogenic effect of coffee husk, coffee silverskin, and cocoa shell extracts (31–500 μg/mL) modulating lipid accumulation, intracellular triglyceride content, extracellular glycerol release, lipase activity, mitochondrial content, citrate synthase (CS) activity, and ATP content in 3T3-L1 adipocytes. Values are expressed as mean (µg/mL) ± SD (*n* = 3). Different letters among rows indicate significant differences (*p* < 0.05) according to ANOVA and Tukey’s multiple range test.

Biomarkers	Coffee Husk		Coffee Silverskin		Cocoa Shell	
	*EC*_30_	*EC*_50_	*EC*_30_	*EC*_50_	*EC*_30_	*EC*_50_
Lipid accumulation	132.7 ± 18.1^a^	309.6 ± 23.8_a_	220.8 ± 35.1^b^	515.3 ± 46.1_b_	388.1 ± 67.4^c^	905.5 ± 88.4_c_
Triglyceride content	64.9 ± 12.4^a^	151.5 ± 30.5_b_	49.6 ± 17.5^a^	115.7 ± 22.3_a_	353.1 ± 64.5^b^	823.8 ± 146.7_c_
Glycerol release	<31.0	<31.0	<31.0	<31.0	36.8 ± 8.4	85.9 ± 4.3
Lipase activity	357.3 ± 73.8^b^	833.6 ± 106.7_b_	210.9 ± 33.4^a^	492.1 ± 48.6_a_	422.2 ± 57.6^b^	985.2 ± 142.9_b_
Mitochondrial content	>500	>500	>500	>500	>500	>500
CS activity	227.6 ± 43.1^b^	531.1 ± 62.4_b_	140.9 ± 22.8^a^	328.9 ± 33.2_a_	247.4 ± 51.4^b^	577.2 ± 74.3_b_
ATP content	>500	>500	251.8 ± 24.0^a^	587.5 ± 35.2_a_	312.4 ± 44.6^b^	728.9 ± 65.1_b_

## Supplementary Materials

The following are available on line at https://www.mdpi.com/2076-3921/8/8/279/s1, Figure S1: Impact of treatments on cell viability of (A) RAW264.7 macrophages, (B) 3T3-L1 preadipocytes, and (C) 3T3-L1 differentiated adipocytes. Cells were treated in the absence (NT) or presence of coffee husk, coffee silverskin, or cocoa shell aqueous extracts (31–500 μg/mL) for 24 h, after which the cell viability was determined using an MTS colorimetric assay. The results are expressed as mean ± SD (*n* = 3). NT: non-treated cells; Figure S2: Heat map including standardized principal component regression (PCR) coefficient of the regression constructed among the phytochemicals identified in coffee husk, coffee silverskin, and cocoa shell aqueous extracts and the potential of the extracts in the different biomarkers of inflammation, oxidative stress, adipogenesis, and insulin resistance; Figure S3: Dendrograms of hierarchical cluster analysis of the treatments of coffee husk (CHE), coffee silverskin (CSE), and cocoa shell (CAE) on the different in vitro models od inflammation, oxidative stress, adipogenesis, and insulin resistance. All the variables in each group of biological activity were included. NT: non-treated cells; Table S1: Anti-inflammatory effect of coffee husk, coffee silverskin, and cocoa shell extracts regulating NO, PGE2, TNF-α, and MCP-1 release in LPS-stimulated RAW264.7 macrophages, and TNF-α, MCP-1, IL-6, adiponectin, intracellular triglyceride content, extracellular glycerol release, and lipase activity CM-stimulated in 3T3-L1 adipocytes. Values are expressed as mean ± SD (*n* = 3). Different letters among rows (same concentration) indicate significant differences (*p* < 0.05) according to ANOVA and Tukey’s multiple range test; Table S2: Protective effect of coffee husk, coffee silverskin, and cocoa shell extracts against oxidative stress and mitochondrial dysfunction regulating ROS and ΔΨm in RAW264.7 macrophages and ROS, mitochondrial O_2_^• ‒^, ΔΨm, mitochondrial content, citrate synthase activity, OCR, and ATP content in 3T3-L1 adipocytes. Values are expressed as mean ± SD (*n* = 3). Different letters among rows (same concentration) indicate significant differences (*p* < 0.05) according to ANOVA and Tukey’s multiple range test; Table S3: Anti-adipogenic effect of coffee husk, coffee silverskin, and cocoa shell extracts modulating lipid accumulation, intracellular triglyceride content, extracellular glycerol release, lipase activity, mitochondrial content, citrate synthase activity, and ATP content in 3T3-L1 adipocytes. Values are expressed as mean ± SD (*n* = 3). Different letters among rows indicate significant differences (*p* < 0.05) according to ANOVA and Tukey’s multiple range test; Table S4: Significantly (*p* < 0.05) up- and down-phosphorylated proteins, expressed as log_2_ (Fold Change), in insulin-resistant 3T3-L1 cells in response to treatment with extracts from coffee husk, coffee silverskin, and cocoa shell.
