# Supplementary material for: Relationship of the Phytochemicals from Coffee and Cocoa By-Products with their Potential to Modulate Biomarkers of Metabolic Syndrome In Vitro
Source: Antioxidants (Basel). 2019 Aug 5;8(8):279. doi: 10.3390/antiox8080279 (PMC6721099; doi:10.3390/antiox8080279)
Supplement: Supplementary file 1 [file antioxidants-08-00279-s001.pdf]

antioxidants

Supplementary Material

Relationship of the Phytochemicals from Coffee and Cocoa By-Products with their Potential to Modulate Biomarkers of Metabolic Syndrome *In Vitro*

Miguel Rebollo-Hernanz [1,2,3], Qiaozhi Zhang [3,4], Yolanda Aguilera [1,2], Maria A. Martín-Cabrejas [1,2], Elvira Gonzalez de Mejia [3*]

[1] Institute of Food Science Research, CIAL (UAM-CSIC), 28049, Madrid, Spain.
[2] Department of Agricultural Chemistry and Food Science, Universidad Autónoma de Madrid, 28049, Madrid, Spain.
[3] Department of Food Science and Human Nutrition, University of Illinois at Urbana-Champaign, IL 61801, United States.
[4] College of Food Science and Biotechnology, Zhejiang Gongshang University, Hangzhou, 310000, China.
* Correspondence: edemejia@illinois; Tel.: +1-217-244-3196 (E.G.d.M.)

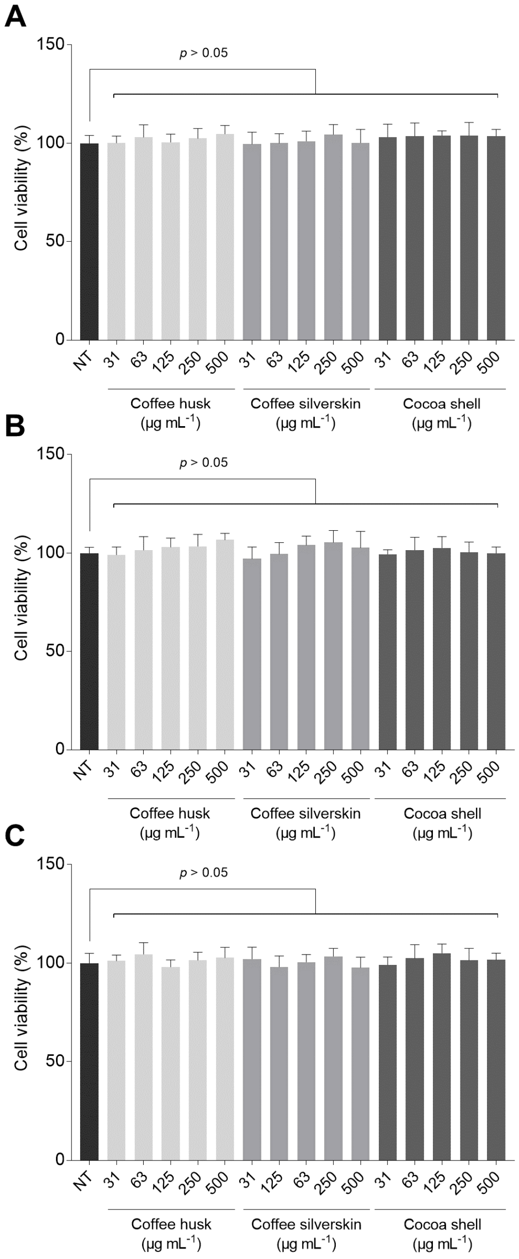

Figure S1. Impact of treatments on cell viability of (**A**) RAW264.7 macrophages, (**B**) 3T3-L1 preadipocytes, and (**C**) 3T3-L1 differentiated adipocytes. Cells were treated in the absence (NT) or presence of coffee husk, coffee silverskin, or cocoa shell aqueous extracts (31-500 μg mL^{-1}) for 24 h, after which, the cell viability was determined using an MTS colorimetric assay. The results are expressed as mean ± SD (n = 3). NT: non-treated cells.

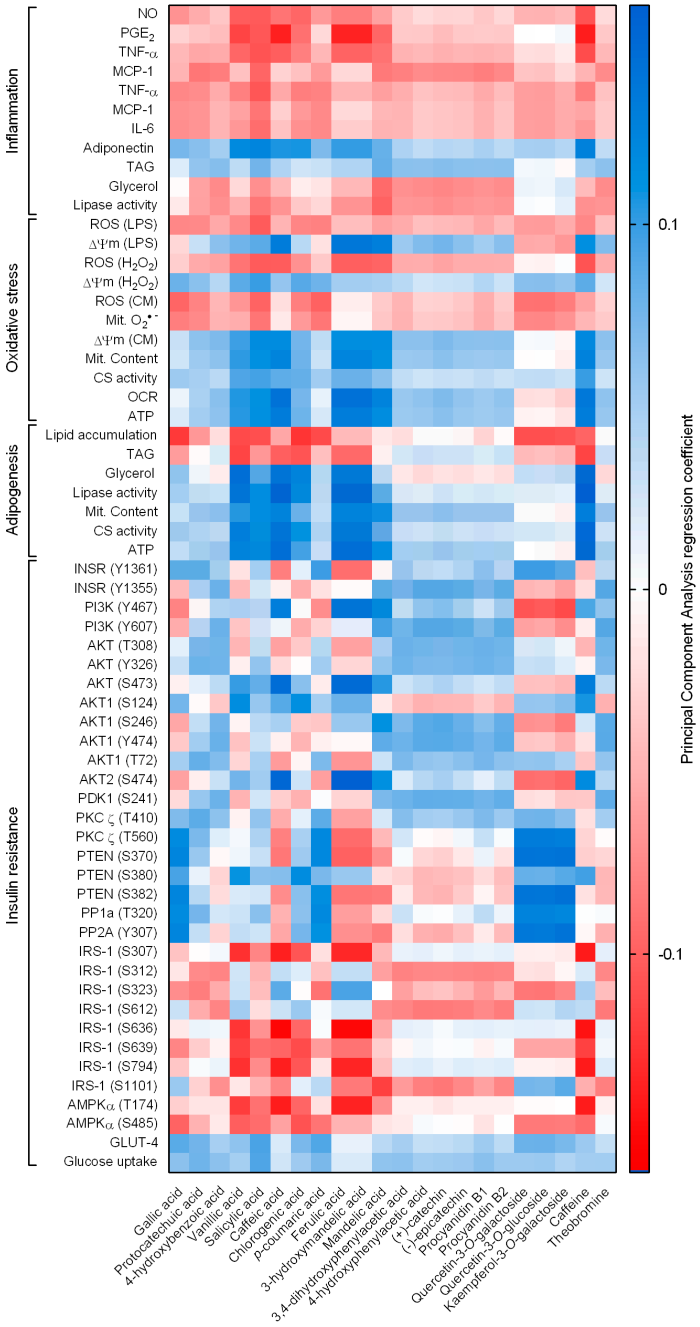

Figure S2. Heat map including standardized principal component regression (PCR) coefficient of the regression constructed among the phytochemicals found in coffee husk, coffee silverskin, and cocoa shell aqueous extracts and the potential of the extracts in the different biomarkers of inflammation, oxidative stress, adipogenesis, and insulin resistance.

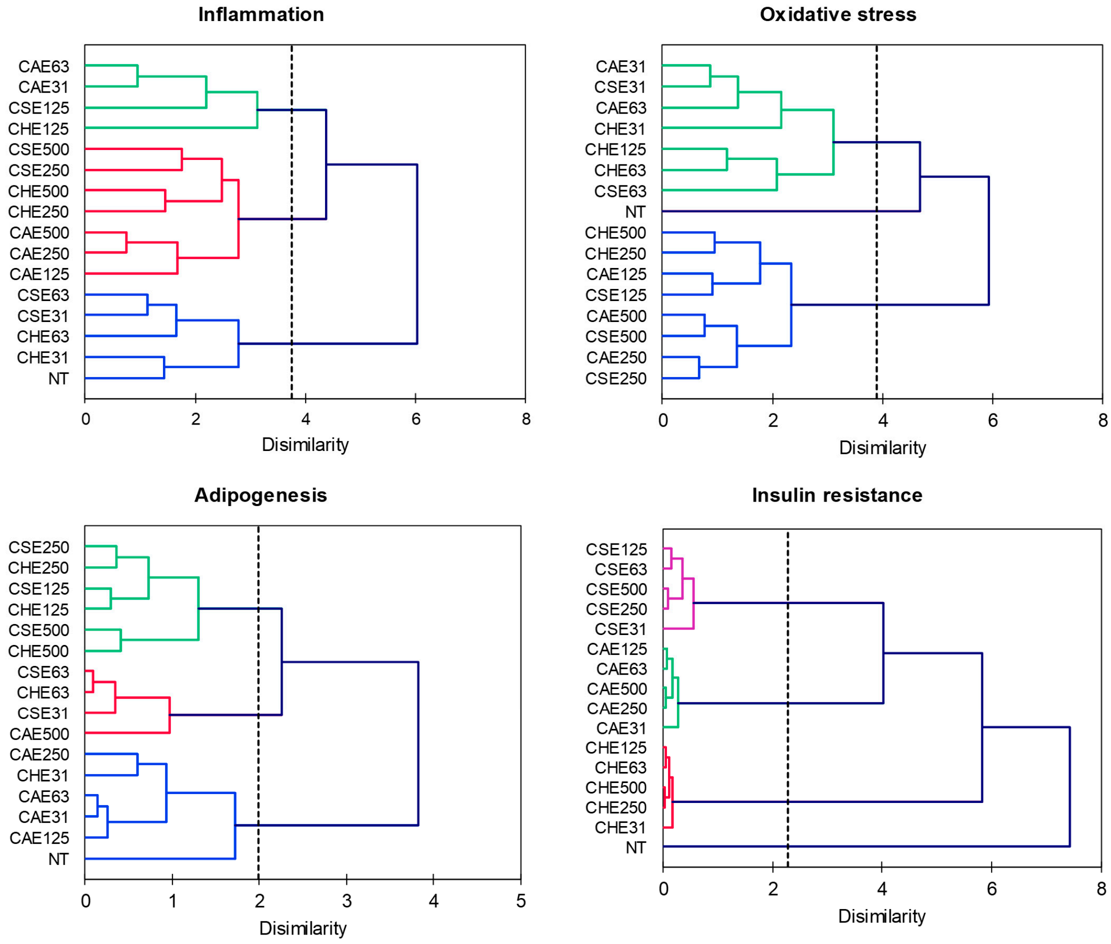

Figure S3. Dendrograms of hierarchical cluster analysis of the treatments of coffee husk (CHE), coffee silverskin (CSE), and cocoa shell (CAE) on the different in vitro models od inflammation, oxidative stress, adipogenesis, and insulin resistance. All the variables in each group of biological activity were included. NT: non-treated cells

Table S1. Anti-inflammatory effect of coffee husk, coffee silverskin, and cocoa shell extracts regulating NO, PGE2, TNF-α, and MCP-1 release in LPS-stimulated RAW264.7 macrophages, and TNF-α, MCP-1, IL-6, adiponectin, intracellular triglyceride content, extracellular glycerol release, and lipase activity CM-stimulated in 3T3-L1 adipocytes. Values are expressed as mean ± SD (n = 3). Different letters among rows (same concentration) indicate significant differences ($p < 0.05$) according to ANOVA and Tukey's multiple range test.

		RAW264.7 macrophages				***3T3-L1 adipocytes***						
		NO release	PGE2 release	TNF-α release	MCP-1 release	TNF-α release	MCP-1 release	IL-6 release	Adiponectin release	TAG content	Glycerol release	Lipase activity
**NT**		100.0 ± 5.3^{z}	100.0 ± 3.9^{z}	100.0 ± 2.7^{z}	100.0 ± 12.2^{z}	100.0 ± 3.4^{z}	100.0 ± 8.2^{z}	100.0 ± 7.1^{z}	0.0 ± 0.6^{z}	0.0 ± 3.9^{z}	100.0 ± 2.5^{z}	100.0 ± 10.8^{z}
**Coffee husk (μg/mL)**	*31*	57.6 ± 5.7$_{a}^{y}$	78.7 ± 2.5$_{a}^{y}$	78.0 ± 3.9$_{b}^{y}$	84.2 ± 2.0$_{b}^{y}$	69.6 ± 5.5$_{a}^{y}$	52.8 ± 3.5$_{a}^{y}$	75.0 ± 0.3$_{a}^{y}$	8.0 ± 1.4$_{b}^{y}$	23.3 ± 6.2$_{a}^{y}$	81.7 ± 7.9$_{b}^{y}$	91.1 ± 2.7$_{b}^{z}$
	63	51.5 ± 6.0$_{a}^{yx}$	69.2 ± 1.6$_{a}^{x}$	71.4 ± 3.9$_{b}^{x}$	72.6 ± 3.9$_{b}^{x}$	57.3 ± 0.2$_{a}^{x}$	46.5 ± 3.2$_{a}^{yx}$	72.9 ± 0.1$_{a}^{x}$	20.6 ± 8.0$_{b}^{x}$	59.9 ± 5.2$_{a}^{x}$	77.1 ± 8.1$_{c}^{y}$	79.0 ± 7.5$_{b}^{y}$
	125	40.2 ± 6.2$_{a}^{x}$	65.4 ± 2.0$_{b}^{x}$	69.1 ± 5.4$_{b}^{x}$	55.0 ± 3.0$_{b}^{w}$	48.2 ± 0.1$_{a}^{w}$	42.9 ± 4.8$_{a}^{x}$	67.9 ± 1.0$_{a}^{w}$	36.0 ± 4.7$_{a}^{w}$	63.2 ± 3.8$_{a}^{x}$	56.3 ± 4.5$_{c}^{x}$	60.6 ± 15.2$_{b}^{yx}$
	250	30.6 ± 3.1$_{a}^{w}$	58.9 ± 3.9$_{c}^{w}$	63.7 ± 6.8$_{a}^{xw}$	49.1 ± 6.1$_{b}^{w}$	47.4 ± 0.4$_{a}^{v}$	38.4 ± 4.7$_{a}^{wx}$	62.6 ± 0.1$_{a}^{v}$	52.3 ± 4.7$_{a}^{v}$	83.2 ± 6.1$_{a}^{w}$	52.1 ± 9.9$_{b}^{x}$	48.2 ± 10.8$_{b}^{xw}$
	500	22.7 ± 3.2$_{a}^{v}$	52.7 ± 0.9$_{c}^{v}$	54.4 ± 4.5$_{b}^{w}$	36.6 ± 6.2$_{b}^{v}$	38.3 ± 1.4$_{a}^{u}$	36.4 ± 3.8$_{b}^{w}$	60.5 ± 0.1$_{a}^{u}$	71.9 ± 4.4$_{a}^{u}$	96.5 ± 6.9$_{a}^{w}$	51.1 ± 7.4$_{b}^{x}$	41.0 ± 3.9$_{c}^{w}$
**EC_{50} (μg/mL)**		73.4 ± 8.9$_{a}$	312.6 ± 48.4$_{b}$	370.4 ± 61.7$_{a}$	203.5 ± 17.7$_{b}$	139.5 ± 21.9$_{a}$	80.0 ± 16.6$_{a}$	> 500	225.4 ± 14.3$_{a}$	60.3 ± 10.0$_{b}$	265.7 ± 42.2$_{c}$	249.8 ± 29.7$_{c}$
**Coffee silverskin (μg/mL)**	*31*	77.0 ± 4.9$_{b}^{y}$	77.4 ± 3.4$_{a}^{y}$	85.9 ± 8.7$_{b}^{y}$	92.0 ± 9.7$_{c}^{z}$	92.0 ± 1.5$_{b}^{y}$	62.9 ± 4.2$_{b}^{y}$	79.0 ± 6.1$_{a}^{y}$	2.1 ± 1.6$_{a}^{z}$	53.2 ± 5.3$_{b}^{y}$	92.9 ± 9.9$_{c}^{y}$	87.7 ± 7.7$_{b}^{z}$
	63	55.4 ± 5.8$_{a}^{x}$	63.9 ± 4.3$_{a}^{x}$	74.1 ± 5.6$_{b}^{yx}$	80.8 ± 8.3$_{b}^{y}$	79.4 ± 5.9$_{b}^{x}$	60.4 ± 5.4$_{b}^{y}$	73.5 ± 1.0$_{a}^{yx}$	10.3 ± 2.8$_{a}^{y}$	63.2 ± 4.8$_{a}^{x}$	69.2 ± 4.0$_{b}^{x}$	72.4 ± 5.6$_{b}^{y}$
	125	45.3 ± 4.1$_{a}^{x}$	50.9 ± 2.2$_{a}^{w}$	71.1 ± 6.6$_{b}^{x}$	71.9 ± 6.3$_{c}^{yx}$	69.3 ± 6.2$_{c}^{xw}$	57.4 ± 4.0$_{b}^{yx}$	72.8 ± 1.1$_{b}^{xw}$	37.5 ± 7.4$_{a}^{x}$	76.5 ± 8.1$_{a}^{w}$	48.6 ± 5.1$_{b}^{w}$	46.7 ± 4.7$_{b}^{x}$
	250	27.4 ± 2.7$_{a}^{w}$	37.2 ± 4.0$_{a}^{v}$	67.1 ± 6.7$_{a}^{x}$	65.9 ± 6.5$_{c}^{xw}$	55.1 ± 6.8$_{a}^{w}$	49.5 ± 5.4$_{b}^{xw}$	69.9 ± 0.9$_{c}^{w}$	58.2 ± 4.7$_{a}^{w}$	86.5 ± 9.6$_{a}^{wv}$	43.5 ± 5.9$_{b}^{wv}$	35.9 ± 2.4$_{b}^{w}$
	500	22.5 ± 1.9$_{a}^{v}$	29.3 ± 2.6$_{a}^{u}$	40.4 ± 6.6$_{a}^{w}$	47.3 ± 5.9$_{b}^{w}$	44.9 ± 1.3$_{b}^{v}$	46.6 ± 4.6$_{c}^{w}$	66.8 ± 1.5$_{b}^{w}$	75.5 ± 4.2$_{a}^{v}$	96.5 ± 10.0$_{a}^{v}$	41.3 ± 4.7$_{b}^{v}$	28.3 ± 2.9$_{b}^{v}$
**EC_{50} (μg/mL)**		97.5 ± 6.5$_{b}$	135.2 ± 9.4$_{a}$	341.8 ± 42.8$_{a}$	420.3 ± 25.0$_{a}$	321.8 ± 24.1$_{c}$	179.3 ± 36.8$_{b}$	> 500	232.9 ± 16.5$_{a}$	32.6 ± 5.5$_{a}$	187.2 ± 25.7$_{b}$	148.0 ± 12.3$_{b}$
**Cocoa shell (μg/mL)**	*31*	56.0 ± 7.2$_{a}^{y}$	91.9 ± 3.3$_{b}^{y}$	68.4 ± 4.1$_{a}^{y}$	70.7 ± 5.6$_{a}^{y}$	87.8 ± 6.9$_{b}^{y}$	62.5 ± 4.7$_{b}^{y}$	77.6 ± 5.4$_{a}^{y}$	1.0 ± 0.5$_{a}^{z}$	97.0 ± 8.6$_{c}^{y}$	39.4 ± 4.6$_{a}^{y}$	41.6 ± 8.6$_{a}^{y}$
	63	52.2 ± 6.2$_{a}^{y}$	78.2 ± 2.9$_{b}^{x}$	66.0 ± 7.5$_{a}^{yx}$	56.7 ± 6.1$_{a}^{x}$	76.7 ± 4.8$_{b}^{y}$	60.5 ± 2.7$_{b}^{y}$	73.5 ± 2.6$_{a}^{y}$	10.9 ± 1.3$_{a}^{y}$	106.8 ± 11.3$_{b}^{y}$	30.7 ± 4.5$_{a}^{y}$	33.2 ± 8.8$_{a}^{yx}$
	125	48.3 ± 4.5$_{a}^{yx}$	64.2 ± 3.0$_{b}^{w}$	61.6 ± 7.5$_{a}^{yx}$	44.9 ± 4.3$_{a}^{w}$	55.4 ± 3.4$_{b}^{x}$	56.1 ± 3.3$_{b}^{y}$	68.8 ± 2.0$_{a}^{x}$	37.1 ± 4.6$_{a}^{x}$	113.8 ± 12.0$_{b}^{y}$	24.6 ± 2.0$_{a}^{x}$	26.6 ± 6.7$_{a}^{x}$
	250	38.7 ± 4.4$_{b}^{xw}$	50.5 ± 1.1$_{b}^{v}$	56.5 ± 9.7$_{a}^{x}$	19.8 ± 9.2$_{a}^{v}$	47.3 ± 6.0$_{a}^{x}$	43.6 ± 2.8$_{b}^{x}$	65.1 ± 0.2$_{b}^{w}$	48.8 ± 5.0$_{a}^{w}$	119.7 ± 11.6$_{b}^{x}$	18.0 ± 2.1$_{a}^{w}$	20.3 ± 8.3$_{a}^{x}$
	500	30.1 ± 7.2$_{a}^{w}$	43.5 ± 3.2$_{b}^{u}$	49.9 ± 5.5$_{ab}^{x}$	0.6 ± 6.2$_{a}^{u}$	33.3 ± 3.7$_{a}^{w}$	28.8 ± 2.8$_{a}^{w}$	59.9 ± 1.1$_{a}^{v}$	68.5 ± 4.3$_{a}^{v}$	134.3 ± 9.0$_{b}^{w}$	0.1 ± 0.0$_{a}^{v}$	0.3 ± 7.0$_{a}^{w}$
**EC_{50} (μg/mL)**		94.0 ± 16.5$_{ab}$	276.4 ± 19.2$_{b}$	498.9 ± 50.3$_{b}$	76.1 ± 8.6$_{a}$	204.9 ± 15.2$_{b}$	132.5 ± 18.8$_{b}$	> 500	268.7 ± 25.1$_{b}$	< 31	26.7 ± 2.8$_{a}$	30.2 ± 4.4$_{a}$

N.D.: not determined

Table S2. Protective effect of coffee husk, coffee silverskin, and cocoa shell extracts against oxidative stress and mitochondrial dysfunction regulating ROS and ΔΨm in RAW264.7 macrophages and ROS, mitochondrial $O_2^{\bullet -}$, ΔΨm, mitochondrial content, citrate synthase activity, OCR, and ATP content in 3T3-L1 adipocytes. Values are expressed as mean ± SD (n = 3). Different letters among rows (same concentration) indicate significant differences ($p < 0.05$) according to ANOVA and Tukey's multiple range test.

		RAW264.7 macrophages				*3T3-L1 adipocytes*						
		**ROS (LPS)**	**ΔΨm (LPS)**	**ROS (H_2O_2)**	**ΔΨm (H_2O_2)**	**ROS**	**Mit. $O_2^{\bullet -}$**	**ΔΨm**	**Mit. content**	**CS activity**	**OCR**	**ATP content**
**NT**		100.0 ± 6.3^{z}	0.0 ± 5.7^{z}	100.0 ± 9.7^{z}	0.0 ± 2.2^{z}	100.0 ± 4.0^{z}	100.0 ± 5.8^{z}	0.0 ± 2.4^{z}	0.0 ± 5.7^{z}	0.0 ± 2.7^{z}	0.0 ± 5.9^{z}	0.0 ± 3.4^{z}
**Coffee husk (μg/mL)**	*31*	$64.0 \pm 5.7_{b}^{y}$	$3.8 \pm 1.7_{a}^{z}$	$69.2 \pm 6.1_{b}^{y}$	$41.2 \pm 7.4_{c}^{y}$	$73.8 \pm 5.6_{a}^{y}$	$81.9 \pm 9.8_{a}^{y}$	$11.0 \pm 3.0_{a}^{y}$	$14.0 \pm 1.8_{a}^{y}$	$75.6 \pm 11.3_{a}^{y}$	$1.1 \pm 5.1_{ab}^{z}$	$11.4 \pm 0.1_{a}^{y}$
	63	$45.8 \pm 4.9_{b}^{x}$	$6.6 \pm 2.8_{a}^{zy}$	$58.3 \pm 3.1_{b}^{x}$	$69.0 \pm 8.0_{c}^{x}$	$60.1 \pm 5.8_{a}^{x}$	$63.8 \pm 5.6_{a}^{x}$	$22.4 \pm 3.6_{a}^{x}$	$44.7 \pm 3.9_{a}^{x}$	$104.7 \pm 8.2_{a}^{x}$	$14.2 \pm 4.4_{ab}^{y}$	$35.3 \pm 1.7_{a}^{x}$
	125	$27.4 \pm 2.9_{a}^{w}$	$9.1 \pm 2.8_{a}^{y}$	$49.3 \pm 3.6_{c}^{w}$	$81.6 \pm 1.4_{b}^{w}$	$47.3 \pm 7.1_{a}^{xw}$	$53.0 \pm 9.3_{a}^{xw}$	$27.1 \pm 3.8_{a}^{x}$	$55.5 \pm 5.2_{a}^{w}$	$114.6 \pm 0.7_{a}^{xw}$	$18.8 \pm 9.7_{a}^{yx}$	$43.3 \pm 1.8_{a}^{w}$
	250	$7.8 \pm 0.2_{a}^{v}$	$12.6 \pm 1.6_{a}^{x}$	$41.7 \pm 2.9_{c}^{v}$	$92.6 \pm 8.7_{b}^{v}$	$39.5 \pm 5.6_{a}^{wv}$	$48.9 \pm 9.3_{a}^{xw}$	$41.0 \pm 4.5_{a}^{w}$	$71.4 \pm 7.4_{a}^{v}$	$114.9 \pm 8.4_{a}^{w}$	$21.0 \pm 11.9_{a}^{yx}$	$51.2 \pm 3.8_{a}^{v}$
	500	$0.6 \pm 0.3_{a}^{u}$	$20.3 \pm 1.8_{a}^{w}$	$27.8 \pm 3.3_{b}^{u}$	$94.3 \pm 8.1_{b}^{v}$	$33.6 \pm 4.0_{a}^{v}$	$47.4 \pm 7.7_{a}^{w}$	$58.5 \pm 5.4_{a}^{v}$	$96.1 \pm 9.0_{a}^{u}$	$146.9 \pm 23.2_{a}^{v}$	$30.1 \pm 13.6_{a}^{x}$	$67.8 \pm 6.2_{a}^{u}$
**EC_{50} (μg/mL)**		$48.2 \pm 2.4_{b}$	> 500	$120.7 \pm 13.5_{c}$	$33.6 \pm 3.7_{a}$	$128.1 \pm 15.2_{a}$	$201.6 \pm 35.0_{a}$	$336.0 \pm 12.5_{b}$	$96.0 \pm 8.6_{b}$	< 31	$931.1 \pm 171.3_{c}$	$188.2 \pm 16.5_{c}$
**Coffee silverskin (μg/mL)**	*31*	$57.8 \pm 5.4_{ab}^{y}$	$13.9 \pm 3.2_{b}^{y}$	$65.5 \pm 2.3_{b}^{y}$	$31.7 \pm 5.7_{b}^{y}$	$78.5 \pm 6.0_{a}^{y}$	$81.4 \pm 5.1_{a}^{y}$	$13.9 \pm 3.1_{a}^{y}$	$31.7 \pm 3.7_{b}^{y}$	$94.6 \pm 10.3_{b}^{y}$	$11.4 \pm 6.3_{b}^{zy}$	$27.8 \pm 4.8_{c}^{y}$
	63	$53.2 \pm 5.6_{b}^{y}$	$20.5 \pm 2.4_{b}^{x}$	$53.4 \pm 4.8_{b}^{x}$	$46.5 \pm 9.1_{b}^{y}$	$73.9 \pm 1.6_{b}^{y}$	$78.5 \pm 2.0_{b}^{yx}$	$37.1 \pm 4.3_{b}^{x}$	$70.7 \pm 5.6_{b}^{x}$	$100.5 \pm 8.7_{a}^{y}$	$23.3 \pm 8.0_{b}^{yx}$	$52.8 \pm 0.6_{b}^{x}$
	125	$39.7 \pm 4.1_{b}^{x}$	$27.3 \pm 3.1_{b}^{w}$	$38.0 \pm 2.5_{b}^{w}$	$61.9 \pm 3.1_{a}^{x}$	$67.1 \pm 8.9_{b}^{yx}$	$71.4 \pm 6.2_{b}^{xw}$	$49.1 \pm 4.9_{b}^{x}$	$90.2 \pm 4.7_{c}^{w}$	$109.2 \pm 15.1_{a}^{yx}$	$31.3 \pm 6.8_{a}^{x}$	$66.5 \pm 1.8_{c}^{w}$
	250	$26.1 \pm 2.5_{b}^{w}$	$35.9 \pm 4.7_{b}^{v}$	$24.3 \pm 2.6_{b}^{v}$	$76.5 \pm 10.6_{a}^{w}$	$59.9 \pm 6.1_{b}^{x}$	$65.3 \pm 8.2_{b}^{w}$	$63.3 \pm 5.6_{b}^{w}$	$107.9 \pm 2.4_{b}^{v}$	$125.8 \pm 1.3_{b}^{x}$	$38.1 \pm 10.2_{ab}^{x}$	$78.3 \pm 3.9_{b}^{v}$
	500	$17.9 \pm 2.1_{b}^{v}$	$51.8 \pm 4.4_{b}^{u}$	$13.8 \pm 2.2_{a}^{u}$	$80.9 \pm 4.5_{a}^{w}$	$54.9 \pm 5.5_{c}^{x}$	$62.0 \pm 6.4_{b}^{w}$	$76.6 \pm 5.4_{b}^{v}$	$140.8 \pm 2.9_{b}^{u}$	$150.8 \pm 11.2_{a}^{w}$	$66.5 \pm 10.8_{b}^{w}$	$108.3 \pm 3.6_{b}^{u}$
**EC_{50} (μg/mL)**		$69.5 \pm 6.6_{c}$	$391.3 \pm 33.0_{a}$	$71.7 \pm 3.2_{b}$	$75.6 \pm 6.6_{b}$	$350.6 \pm 56.9_{b}$	> 500	$135.3 \pm 9.2_{a}$	$43.2 \pm 6.8_{a}$	< 31	$291.8 \pm 34.0_{a}$	$59.4 \pm 7.6_{a}$
**Cocoa shell (μg/mL)**	*31*	$50.7 \pm 5.1_{a}^{y}$	$23.8 \pm 3.2_{c}^{y}$	$55.0 \pm 6.2_{a}^{y}$	$12.7 \pm 9.1_{a}^{y}$	$75.0 \pm 3.9_{a}^{y}$	$72.4 \pm 8.5_{a}^{y}$	$14.6 \pm 3.2_{a}^{y}$	$12.1 \pm 5.5_{a}^{y}$	$95.0 \pm 8.0_{b}^{zy}$	$0.6 \pm 1.8_{a}^{z}$	$19.8 \pm 5.9_{b}^{y}$
	63	$38.0 \pm 3.5_{a}^{x}$	$25.8 \pm 2.1_{c}^{y}$	$44.6 \pm 3.2_{a}^{x}$	$30.2 \pm 2.4_{a}^{x}$	$68.4 \pm 4.8_{ab}^{y}$	$63.2 \pm 6.1_{a}^{yx}$	$22.3 \pm 3.6_{a}^{x}$	$36.8 \pm 5.5_{a}^{x}$	$109.1 \pm 8.4_{a}^{yw}$	$11.8 \pm 5.3_{a}^{zy}$	$31.6 \pm 4.3_{a}^{x}$
	125	$26.0 \pm 3.0_{a}^{w}$	$33.7 \pm 3.9_{c}^{x}$	$31.5 \pm 4.1_{a}^{w}$	$63.6 \pm 9.2_{a}^{w}$	$58.0 \pm 4.2_{ab}^{x}$	$59.8 \pm 1.3_{a}^{x}$	$38.9 \pm 4.4_{b}^{w}$	$76.5 \pm 6.6_{b}^{w}$	$116.5 \pm 10.5_{a}^{w}$	$25.3 \pm 10.3_{ab}^{yx}$	$55.7 \pm 3.0_{b}^{w}$
	250	$8.1 \pm 6.2_{a}^{v}$	$40.8 \pm 4.8_{b}^{xw}$	$19.9 \pm 2.0_{a}^{v}$	$79.3 \pm 1.2_{a}^{w}$	$45.7 \pm 4.1_{a}^{w}$	$51.8 \pm 6.6_{a}^{w}$	$55.0 \pm 5.2_{b}^{v}$	$109.2 \pm 7.3_{b}^{v}$	$124.4 \pm 10.4_{ab}^{wv}$	$39.7 \pm 5.9_{b}^{x}$	$77.7 \pm 8.0_{b}^{v}$
	500	$0.5 \pm 1.6_{a}^{u}$	$46.0 \pm 5.3_{b}^{w}$	$13.1 \pm 3.1_{a}^{u}$	$89.9 \pm 1.5_{b}^{v}$	$41.3 \pm 4.1_{b}^{w}$	$49.2 \pm 4.5_{a}^{w}$	$96.6 \pm 4.5_{c}^{u}$	$144.8 \pm 7.2_{b}^{u}$	$135.4 \pm 3.1_{a}^{v}$	$61.8 \pm 7.6_{b}^{w}$	$105.7 \pm 8.0_{b}^{u}$
**EC_{50} (μg/mL)**		$34.7 \pm 2.7_{a}$	$334.9 \pm 52.8_{a}$	$46.6 \pm 3.5_{a}$	$97.8 \pm 12.4_{c}$	$192.3 \pm 23.4_{a}$	$221.2 \pm 40.4_{a}$	$161.5 \pm 22.9_{a}$	$74.9 \pm 12.1_{b}$	< 31	$368.8 \pm 35.9_{b}$	$91.9 \pm 14.3_{b}$

N.D.: not determined

Table S3. Anti-adipogenic effect of coffee husk, coffee silverskin, and cocoa shell extracts modulating lipid accumulation, intracellular triglyceride content, extracellular glycerol release, lipase activity, mitochondrial content, citrate synthase activity, and ATP content in 3T3-L1 adipocytes. Values are expressed as mean ± SD ($n = 3$). Different letters among rows indicate significant differences ($p < 0.05$) according to ANOVA and Tukey's multiple range test.

		Lipid accumulation	Triglyceride content	Glycerol release	Lipase activity	Mitochondrial content	CS activity	ATP content
**NT**		100.0 ± 3.6^{z}	100.0 ± 4.2^{z}	0.0 ± 7.6^{z}	0.0 ± 2.2^{z}	0.0 ± 4.4^{z}	0.0 ± 3.9^{z}	0.0 ± 3.7^{z}
**Coffee husk (µg/mL)**	*31*	$95.9 \pm 3.8_{b}^{y}$	$63.1 \pm 1.8_{b}^{y}$	$71.1 \pm 3.5_{b}^{y}$	$7.6 \pm 1.6_{a}^{y}$	$1.0 \pm 2.8_{a}^{z}$	$9.0 \pm 3.5_{a}^{y}$	$2.9 \pm 1.9_{a}^{z}$
	63	$81.1 \pm 3.4_{a}^{x}$	$55.9 \pm 3.1_{a}^{w}$	$99.6 \pm 2.5_{b}^{x}$	$15.1 \pm 5.8_{a}^{x}$	$5.9 \pm 1.9_{a}^{z}$	$20.3 \pm 4.1_{a}^{x}$	$8.7 \pm 1.7_{a}^{y}$
	125	$66.7 \pm 4.2_{a}^{w}$	$53.2 \pm 3.9_{a}^{wv}$	$111.1 \pm 0.8_{b}^{w}$	$18.4 \pm 6.6_{a}^{x}$	$8.4 \pm 1.5_{a}^{y}$	$24.9 \pm 4.4_{a}^{x}$	$11.9 \pm 2.6_{a}^{y}$
	250	$56.1 \pm 7.2_{a}^{v}$	$48.9 \pm 0.3_{b}^{v}$	$129.9 \pm 2.1_{b}^{v}$	$23.5 \pm 8.8_{a}^{xw}$	$12.1 \pm 4.0_{a}^{yx}$	$31.5 \pm 3.7_{a}^{w}$	$17.6 \pm 3.0_{a}^{x}$
	500	$40.9 \pm 6.8_{a}^{u}$	$44.2 \pm 0.4_{b}^{u}$	$162.6 \pm 19.0_{b}^{u}$	$31.7 \pm 4.7_{a}^{w}$	$17.6 \pm 2.9_{a}^{x}$	$40.9 \pm 4.2_{a}^{v}$	$27.5 \pm 3.4_{a}^{x}$
**EC_{50} (µg/mL)**		$309.6 \pm 23.8_{a}$	$151.5 \pm 30.5_{b}$	$4.1 \pm 1.7_{a}$	$833.6 \pm 106.7_{b}$	> 500	$531.1 \pm 62.4_{b}$	$1193.0 \pm 98.7_{c}$
**Coffee silverskin (µg/mL)**	*31*	$89.6 \pm 3.2_{a}^{y}$	$56.9 \pm 3.6_{a}^{y}$	$192.5 \pm 12.3_{c}^{y}$	$13.5 \pm 5.8_{a}^{y}$	$4.8 \pm 2.4_{a}^{z}$	$18.1 \pm 2.6_{b}^{y}$	$7.3 \pm 2.4_{b}^{y}$
	63	$84.7 \pm 1.8_{a}^{x}$	$55.4 \pm 2.5_{a}^{y}$	$204.2 \pm 2.9_{c}^{y}$	$16.3 \pm 6.0_{a}^{y}$	$6.7 \pm 2.7_{a}^{z}$	$22.0 \pm 3.2_{a}^{y}$	$9.8 \pm 1.3_{a}^{y}$
	125	$75.1 \pm 5.0_{ab}^{w}$	$49.8 \pm 6.2_{a}^{yx}$	$225.8 \pm 1.6_{c}^{x}$	$22.3 \pm 8.2_{a}^{y}$	$11.2 \pm 3.7_{b}^{y}$	$30.0 \pm 3.6_{a}^{x}$	$16.2 \pm 2.0_{b}^{x}$
	250	$68.9 \pm 4.5_{b}^{w}$	$44.3 \pm 2.4_{a}^{x}$	$261.3 \pm 1.4_{c}^{w}$	$31.5 \pm 14.4_{a}^{yw}$	$17.5 \pm 3.9_{b}^{y}$	$40.7 \pm 4.2_{b}^{w}$	$27.3 \pm 3.9_{b}^{w}$
	500	$55.5 \pm 6.7_{b}^{v}$	$37.0 \pm 1.8_{a}^{w}$	$320.0 \pm 11.3_{c}^{v}$	$45.6 \pm 5.4_{b}^{w}$	$26.6 \pm 4.5_{b}^{x}$	$54.5 \pm 6.1_{b}^{v}$	$48.0 \pm 5.2_{c}^{v}$
**EC_{50} (µg/mL)**		$515.3 \pm 46.1_{b}$	$115.7 \pm 22.3_{a}$	< 31	$492.1 \pm 48.6_{a}$	> 500	$328.9 \pm 33.2_{a}$	$587.5 \pm 35.2_{a}$
**Cocoa shell (µg/mL)**	*31*	$94.7 \pm 2.2_{b}^{y}$	$88.1 \pm 4.5_{c}^{y}$	$43.6 \pm 6.1_{a}^{y}$	$6.5 \pm 3.5_{a}^{y}$	$3.9 \pm 1.9_{a}^{z}$	$5.9 \pm 2.0_{a}^{z}$	$4.2 \pm 1.7_{a}^{z}$
	63	$90.2 \pm 2.3_{b}^{x}$	$82.9 \pm 2.5_{b}^{yx}$	$48.0 \pm 9.2_{a}^{yx}$	$15.4 \pm 3.4_{a}^{x}$	$12.4 \pm 2.6_{b}^{y}$	$16.6 \pm 3.5_{a}^{y}$	$9.5 \pm 2.5_{a}^{y}$
	125	$84.7 \pm 6.4_{b}^{xw}$	$81.3 \pm 2.2_{b}^{x}$	$53.9 \pm 8.4_{a}^{yx}$	$18.4 \pm 4.4_{a}^{xw}$	$16.5 \pm 4.3_{b}^{yx}$	$25.6 \pm 3.7_{a}^{x}$	$20.0 \pm 3.1_{c}^{x}$
	250	$77.8 \pm 5.6_{c}^{wv}$	$75.1 \pm 5.1_{c}^{xw}$	$62.6 \pm 10.7_{a}^{xw}$	$22.1 \pm 4.7_{a}^{xw}$	$19.3 \pm 3.9_{b}^{x}$	$32.4 \pm 4.8_{a}^{w}$	$28.2 \pm 3.4_{b}^{w}$
	500	$67.5 \pm 7.8_{c}^{v}$	$72.3 \pm 4.6_{c}^{w}$	$75.9 \pm 5.5_{a}^{w}$	$26.3 \pm 4.3_{a}^{w}$	$23.3 \pm 3.2_{b}^{x}$	$37.3 \pm 4.0_{a}^{v}$	$35.1 \pm 4.0_{b}^{v}$
**EC_{50} (µg/mL)**		$905.5 \pm 88.4_{c}$	$823.8 \pm 146.7_{c}$	$85.9 \pm 4.3_{b}$	$985.2 \pm 142.9_{b}$	> 500	$577.2 \pm 74.3_{b}$	$728.9 \pm 65.1_{b}$

N.D.: not determined

Table S4. Significantly ($p < 0.05$) up- and down-phosphorylated proteins, expressed as $\log_2$ (Fold Change), in insulin-resistant 3T3-L1 cells in response to treatment with extracts from coffee husk, coffee silverskin, and cocoa shell.

Protein	Phosphosite	Effect of phosphorylation	$\log_2$ (Fold Change)		
			Coffee husk	Coffee silverskin	Cocoa shell
Insulin receptor signaling					
Caveolin-1	Y14	Induces activity	1.06	0.90	1.12
INSR	Y1361	Induces activity	2.08	0.65	2.07
	Y1355	Induces activity	0.53	1.26	3.14
IRS-1	S307	Inhibits molecular association	–0.92	–1.52	–0.52
	S312	Inhibits molecular association	–0.47	–	–1.15
	S323	Inhibits activity	–0.51	–	–0.58
	S612	Inhibits activity	–	–	–0.90
	S639	Inhibits molecular association	–2.22	–2.23	–1.11
	S794	Inhibits molecular association	–0.76	–1.27	–0.40
	S1101	Inhibits activity	–	–0.49	–0.77
GAB1	T627	Induces molecular association	–1.51	–1.06	–1.23
	T659	Induces molecular association	–	0.48	1.88
GAB2	S159	Induces molecular association	0.57	–	–
SHC	Y349	Induces activity	0.83	–	0.98
	Y427	Induces activity	0.51	0.62	–
SHP-2	T542	Induces molecular association	–1.64	–0.37	–0.40
	Y580	Induces molecular association	–	–0.44	–
PI3K-AKT-PKB signaling					
4E-BP1	T45	Inhibits molecular association	–1.15	–0.74	0.44
	S65	Inhibits molecular association	–0.89	0.32	–
	T70	Inhibits activity/molecular association	–	–1.84	0.48
ACLY	S454	Induces activity	–0.44	–	–
AKT	T308	Induces activity	1.24	0.33	2.06
	Y326	Induces activity	0.35	–	0.53
	S473	Induces activity	0.36	–0.59	0.47
AKT1	S124	Induces activity	1.39	1.40	0.37
	S246	Induces activity	–	1.02	1.59
	Y474	Induces activity	0.47	0.68	1.90
	T72	Induces activity	1.37	0.54	1.70
AKT2	S474	Induces activity	–	0.86	0.62
BAD	S112	Inhibits molecular association	0.79	–	–
	S134	Inhibits molecular association	0.46	–	0.92
	S136	Inhibits molecular association	1.00	0.46	1.05
	S155	Inhibits molecular association	0.40	0.73	0.52

	S91/18	Inhibits molecular association	1.46	–	1.43
EIF2A	S51	Inhibits activity	–	–0.64	–
EIF4E	S209	Inhibits molecular interaction	–0.43	–1.52	–
EIF4G	S1108	Inhibits activity	–0.65	–0.32	2.08
FKHR	S256	Inhibits molecular association	0.36	1.44	–
	S319	Inhibits molecular association	0.53	0.75	0.87
FKHRL1	S253	Inhibits activity	0.75	0.58	–
FOXO1/3/4-PAN	T24/32	Inhibits activity	–	–	0.52
FOXO1A	S329	Inhibits activity	0.59	–	0.71
GSK3α	S21	Inhibits activity	–	–0.51	0.36
GSK3α/β	T216/279	Induces activity	–	–1.33	–
GSK3β	S9	Inhibits activity	–0.65	–0.89	–
HSL	S552	Induces activity	–1.09	–	–
	S563	Induces activity	–1.09	–	–
	S554	Inhibits activity	–	1.12	1.86
mTOR	T2446	Induces activity	–	–	–0.59
	S2448	Induces activity	–0.75	–	–
p70S6K	T229	Induces activity	–0.33	–	–0.52
	S371	Induces activity	–	–0.54	–
	S411	Induces activity	0.39	–	–
	S418	Induces activity	–	1.42	–
	T421	Induces activity	–2.15	–2.29	–1.07
	S424	Induces activity	–0.92	–1.44	–0.41
	T389	Induces activity	–	–	–1.15
p70S6Kβ	S423	Induces activity	0.75	0.44	0.73
PDK1	S241	Induces activity	–	–	1.05
PI3Kα/γ	T199/467	Induces activity	–	1.43	1.29
PI3Kα	T607	Induces activity	–	0.61	1.59
PKC ζ	T410	Induces activity	0.66	–	0.75
	T560	Induces activity	0.72	–	0.45
PP1α	T320	Inhibits activity	3.89	2.61	3.34
PP2Aα	T307	Inhibits activity	–3.72	–1.18	–
PTEN	S370	Inhibits activity	3.00	–	1.60
	S380	Inhibits activity	1.68	1.52	0.69
	S380/382/383	Inhibits activity	3.34	–	0.52
TSC2	S939	Inhibits activity	–1.74	–2.11	–0.54
AMPK signaling					
AMPK1	T174	Induces activity	–0.92	–1.51	–0.80
AMPK1/2	S485/491	Inhibits activity	–1.07	–0.82	–0.63
AMPKβ	S182	Regulates cellular localization	–0.77	–	–
LKB1	T189	Inhibits activity	–1.70	–0.49	–0.65

LKB1	S428	Induces activity	0.62	–	–
PKA	T197	Induces activity	–	0.93	–
MAPK signaling					
c-Raf	S296	Inhibits activity	1.16	–	0.73
	S43	Inhibits activity	0.37	0.54	0.53
CrkII	Y221	Inhibits activity	2.73	0.58	2.36
GRB10	T67	Inhibits molecular association	–	–	1.15
ERK1	T202	Induces activity	–	–1.19	–
	Y204	Induces activity	–0.39	–0.66	–
ERK3	S189	Induces molecular association	0.75	–	0.57
MEK1	S217	Induces activity	–0.67	–1.31	–
	S221	Induces activity	–	–0.48	–0.37
	T286	Inhibits activity	0.59	–	0.89
	T291	Inhibits activity	0.90	–	1.56
	S298	Induces activity	–1.56	–0.32	–
MEK2	T394	Inhibits activity	0.84	0.44	1.29
Ras-GRF	S916	Induces activity	0.49	–	0.99
IKK-NF-κB signaling					
IKKα	T23	Induces activity	–	–0.65	–0.78
IKKα/β	S180/181	Induces activity	–1.38	–0.32	–0.64
IKKγ	S31	Induces transcription	–	–0.71	–0.54
	S85	Induces activity	–1.31	–	–1.15
iNOS	Expression		–0.78	–	–
PKC θ	T538	Induces activity	–2.26	–1.64	–1.38
	S676	Induces activity	–0.39	–0.48	–
TNFR1	Expression		–1.14	–0.93	–
TNFR2	Expression		–	–	–0.38
